# PrimerROC: accurate condition-independent dimer prediction using ROC analysis

**DOI:** 10.1038/s41598-018-36612-9

**Published:** 2019-01-18

**Authors:** Andrew D. Johnston, Jennifer Lu, Ke-lin Ru, Darren Korbie, Matt Trau

**Affiliations:** 1 Centre for Personalized NanoMedicine, The University of Queensland, St Lucia, 4072 QLD Australia; 2 Australian Institute for Bioengineering and Nanotechnology, The University of Queensland, St Lucia, 4072 QLD Australia; 3 School of Chemistry and Molecular Biosciences, The University of Queensland, St Lucia, 4072 QLD Australia

## Abstract

To-date systematic testing and comparison of the accuracy of available primer-dimer prediction software has never been conducted, due in part to a lack of tools able to measure the efficacy of Gibbs free energy (ΔG) calculations at predicting dimer formation in PCR. To address this we have developed a novel online tool called PrimerROC (www.primer-dimer.com/roc/), which uses epidemiologically-based Receiver Operating Characteristic (ROC) curves to assess dimer prediction accuracy. Moreover, by integrating PrimerROC with our PrimerDimer prediction software we can determine a ΔG-based dimer-free threshold above which dimer formation is predicted unlikely to occur. Notably, PrimerROC determines this cut-off without any additional information such as salt concentration or annealing temperature, meaning that our PrimerROC method is an assay and condition independent prediction tool. To demonstrate the broad utility of PrimerROC we assessed the performance of seven publically available primer design and dimer analysis tools using a dataset of over 300 primer pairs. We found that our PrimerROC/PrimerDimer software consistently outperforms these other tools and can achieve predictive accuracies greater than 92%. To illustrate its predictive power this method was used in multiplex PCR design to successfully generate four resequencing assays containing up to 126 primers with no observable primer-primer amplification artefacts.

## Introduction

Primer design software seeks to maximize product yield and minimize off-target amplification, and a key component of this is the prevention of the primer-primer interaction artefacts known as primer-dimers. Primer-dimers are off-target amplification artefacts formed by primer-primer binding and subsequent elongation. These primer-primer interactions can competitively inhibit binding to target DNA, remove primers from the reaction pool, and exhaust deoxynucleotides—all of which result in reduced amplification efficiency and suboptimal product yields^1^. Thus, accurate dimer prediction algorithms are of great value when designing primers for polymerase-based applications, such as DNA sequencing, polymerase chain reaction (PCR), and a variety of isothermal amplification methods. Accurate prediction is especially important in applications such as real-time PCR, where poor amplification efficiency and off-target artefacts interfere with accurate quantification; as well as multiplexing, where the potential for dimer formation increases polynomially, according to the function (n2 + n)/2, with each primer (n) added to the multiplex reaction^2^.

Numerous primer design programs use the change in Gibbs free energy (ΔG) resulting from primer hybridization as an absolute indicator of dimer formation^3–7^. However, in our assessment of publically available tools, each program calculates ΔG differently, and to-date no studies have empirically demonstrated the efficacy of ΔG in general—or specific algorithms in particular—at predicting primer-dimer formation. While some of these programs offer the ability to alter temperature and PCR component concentrations for ΔG calculations (i.e. Mg++, oligos, dNTPs and monovalent cations), it is unclear how this aids users in determining the likelihood that a particular ΔG value will result in the formation of dimer artefacts. As such, a method for accurate dimer prediction which implicitly normalizes assay-specific prediction values for dimer formation based on a user’s unique conditions would be of significant use to the field. In response to this, we have therefore developed PrimerROC— an application that employs receiver operating characteristic (ROC) curves in a novel way to test predictive power and provide measures of dimer likelihood (sensitivity and specificity) regardless of the unique PCR conditions used.

ROC curves plot the performance of a diagnostic marker as a binary classifier. Each marker value is set as a discrimination threshold, the cut-off below which values are classified as condition positive and above which values are classified as condition negative. The true positive rate (sensitivity) and false positive rate (1-specificity) at each threshold value are then plotted against one another to create the ROC curve. The area under the curve (AUC) provides a measure of overall predictive accuracy (1 being perfect and 0.5 is no better than chance), and ROC curves are regularly used in clinical epidemiology to quantify the accuracy of medical diagnostic tests^8^. Here we use PrimerROC analyses to evaluate and iteratively improve the performance of our ΔG-based dimer-prediction algorithm, PrimerDimer. We then integrate PrimerDimer into PrimerROC to produce an online tool that allows users to distinguish between dimer-forming and dimer-free primer pairs to a high degree of accuracy (>92%). Performance is then compared to seven other commonly used primer design/analysis tools and PrimerROC consistently showed superior predictive power and greater ability to produce dimer-free discrimination thresholds across a variety of primer lengths. Finally, we demonstrate that the high accuracy of PrimerDimer and the dimer-free threshold derived from PrimerROC analysis can be used to prevent dimer formation in highly multiplexed PCR/resequencing reactions.

## Results and Discussion

### Primer-dimer prediction

**Figure 1:**
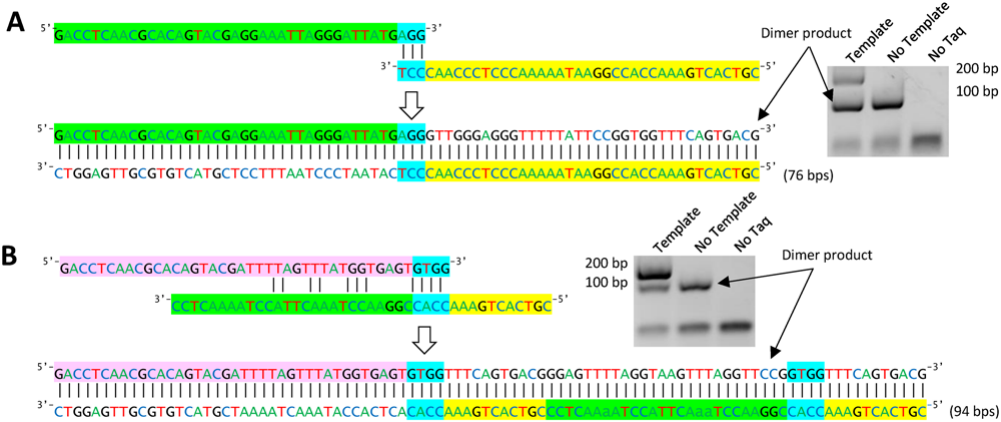
Dimer artefact sequencing used to develop accurate dimer prediction. (**A**,**B**) Sequence data of primer pairs with high concentration dimer artefacts matched to predicted dimer structures. Artefact sequences are arranged in compliments and juxtaposed with their appearance in gel electrophoresis. (**A**) Thermodynamically stable perfect complementarity spanning both 3′ ends, and (**B**) thermodynamically stable complementarity at single 3′ end. Elongation from a single end often results in a duplication of the complementary structure along with the 5′ overhang, as seen in the blue and yellow highlights. Gel images were cropped from full gels in Supplementary Fig. S12.

**Figure 2:**
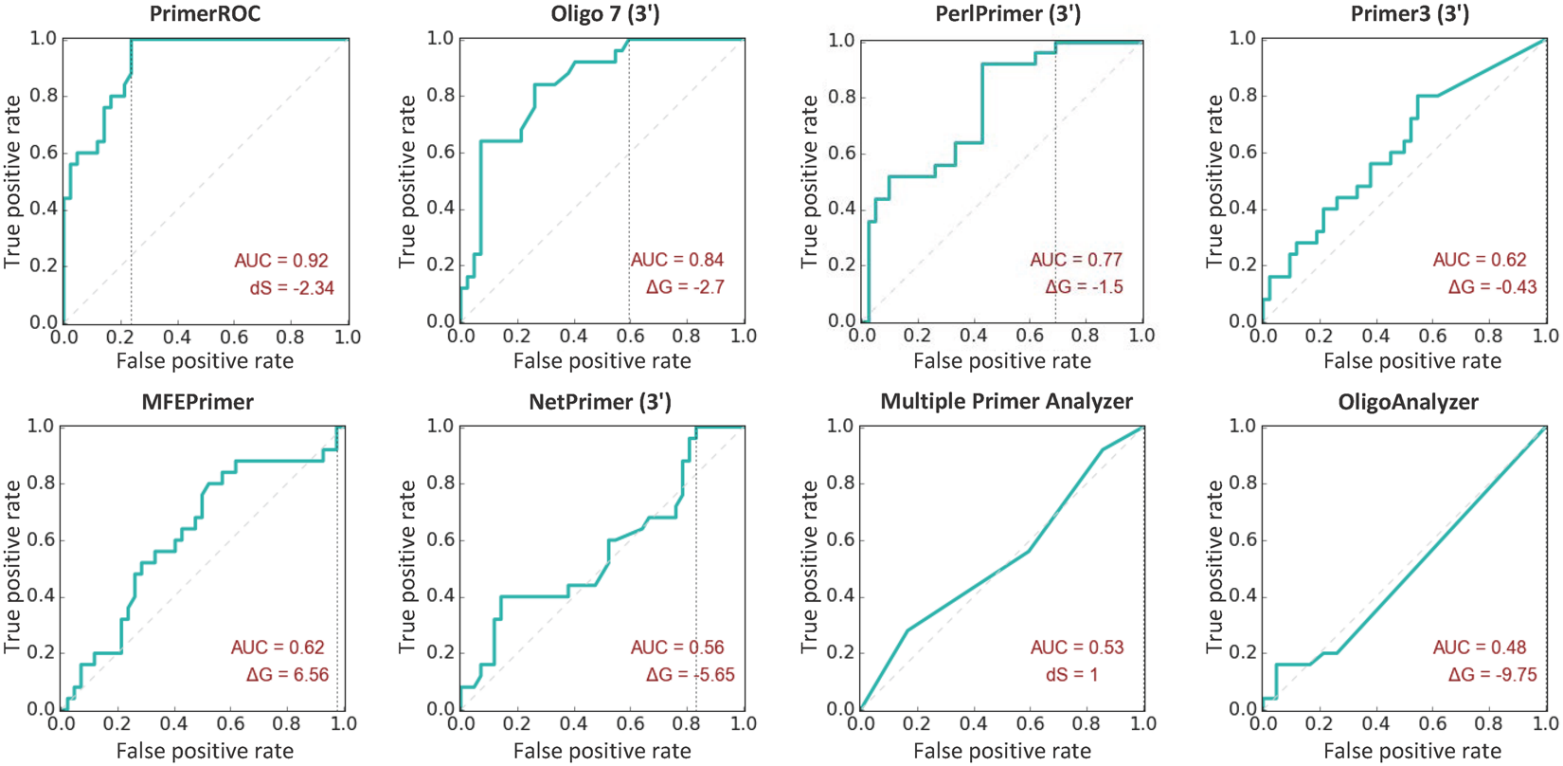
Receiver operator characteristic (ROC) curves of 20 base pair fusion primer set used to measure the predictive performance of dimer algorithms. Included are PrimerROC and the seven freely available dimer prediction algorithms analysed. Curves display the accuracy of dimer prediction as measured by area under the curve (AUC), and dimer score (dS/ΔG) at the dimer-free threshold (the point where zero dimer-forming primer pairs are misclassified as dimer-free). ROC analysis was used to assess and iteratively improve the dimer prediction performance of the PrimerDimer algorithm.

To begin development on our dimer prediction algorithm PrimerDimer, a set of primer-dimer artefacts were first sequenced to assess the types of primer-primer interactions that cause dimer formation (Supplementary Fig. S1). These sequences confirm what other studies have previously stipulated—that the primer-primer interactions of most concern contain stable complements at the 3′ ends (Fig. 1A), allowing for polymerase binding and elongation^1,9,10^. Surprisingly, both 3′ ends do not have to form a continuous stable structure for exponential amplification to occur. Within the sequenced dimers, stable structures at a single 3′ end regularly formed amplification artefacts of high concentrations, although it was also observed that 5′ overhangs on the side of the stable structure were often duplicated in the resulting dimer artefact (Fig. 1B). Based on these observations an algorithm to predict dimer formation was created and then iteratively improved using ROC analyses to measure the predictive accuracy of each new version (Fig. 2). These analyses were performed using sets of primer pairs in which dimer formation was empirically determined via gel electrophoresis of PCR product. Four primer sets (Supplementary Table 1) with different lengths and 5′ fusion sequences (i.e., non-template sequence added to the 5′ end of the oligonucleotide – a method commonly used for library construction) were used—3 (Supplementary Fig. S2) and 20 (Supplementary Fig. S3) base pair fusion primer sets for development of the PrimerDimer algorithm, as well as 2 (Supplementary Fig. S4) and 14 (Supplementary Fig. S5) base pair fusion primer sets to validate the final algorithm and determine the efficacy of the algorithm in different experimental setups.

**Figure 3:**
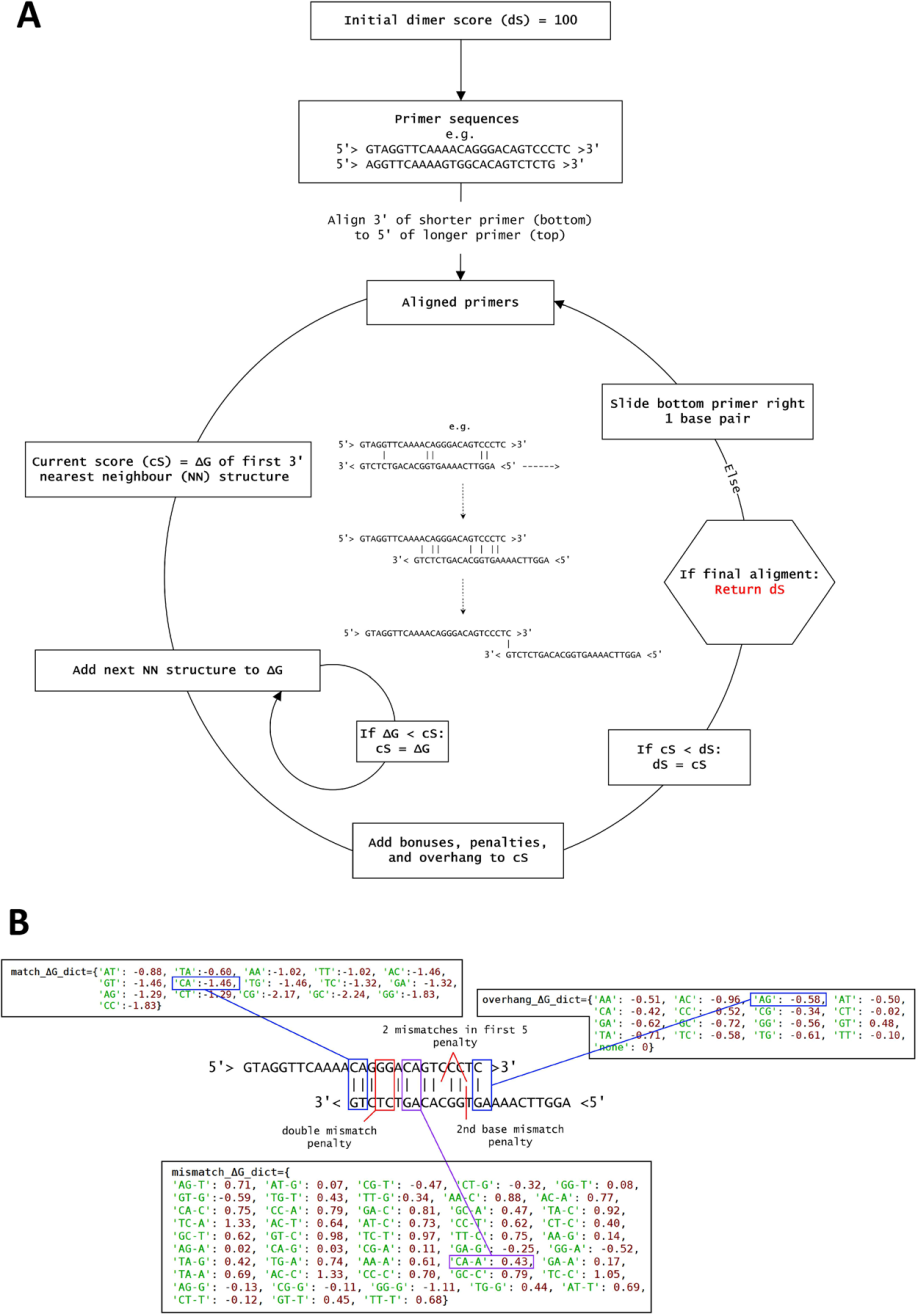
How PrimerDimer calculates dimer score. (**A**) Flowchart detailing the steps involved in calculating dimer score of a primer pair. For each primer pair, PrimerDimer starts with a dimer score of 100 (an arbitrary value greater than the maximum dimer score of 0). The 3′ of the shorter primer (bottom) is aligned to the 5′ of the longer primer (top) and the bottom then slides along the top to produce all possible primer-primer alignments with 5′ overhangs. For each alignment, a score is calculated based on nearest-neighbour (NN) parameters, as well as bonus values and penalties for structures that are more or less conducive to dimer formation, polymerase binding, and transcription initiation. The most negative score from all alignments is returned as the dimer score (dS). (**B**) Example alignment of primer pair, including the dictionaries of ΔG values (kcal/mol) for nearest-neighbour duplexes (match_ ΔG_dict), single-mismatches (mismatch_ ΔG_dict), and 5′ overhangs (overhang_ ΔG_dict), as well as penalties used to calculate the example structure’s score.

The PrimerDimer algorithm works by aligning the 5′ end of the longer primer to the 3′ end of the shorter primer, thus forming a structure with a single 3′ overhang (except with primers of equal length). The shorter primer slides along the longer primer to form all possible dimer structures with 5′ overhangs. The ΔG of each possible dimer structure is calculated using nearest-neighbour parameters for duplexes^11^, single mismatches^12–16^, and 5′ overhangs of bases at the 3′ ends^17^, with each end treated independently. Bonus values and penalties are then added for structures that are more or less conducive to dimer formation, polymerase binding, and transcription initiation. This analysis is performed for the three possible primer-primer pairings within forward and reverse primer sets (1 hetero- and 2 homo-pairs), after which the most negative ΔG-based value is then returned as the dimer score (Fig. 3).

**Figure 4:**
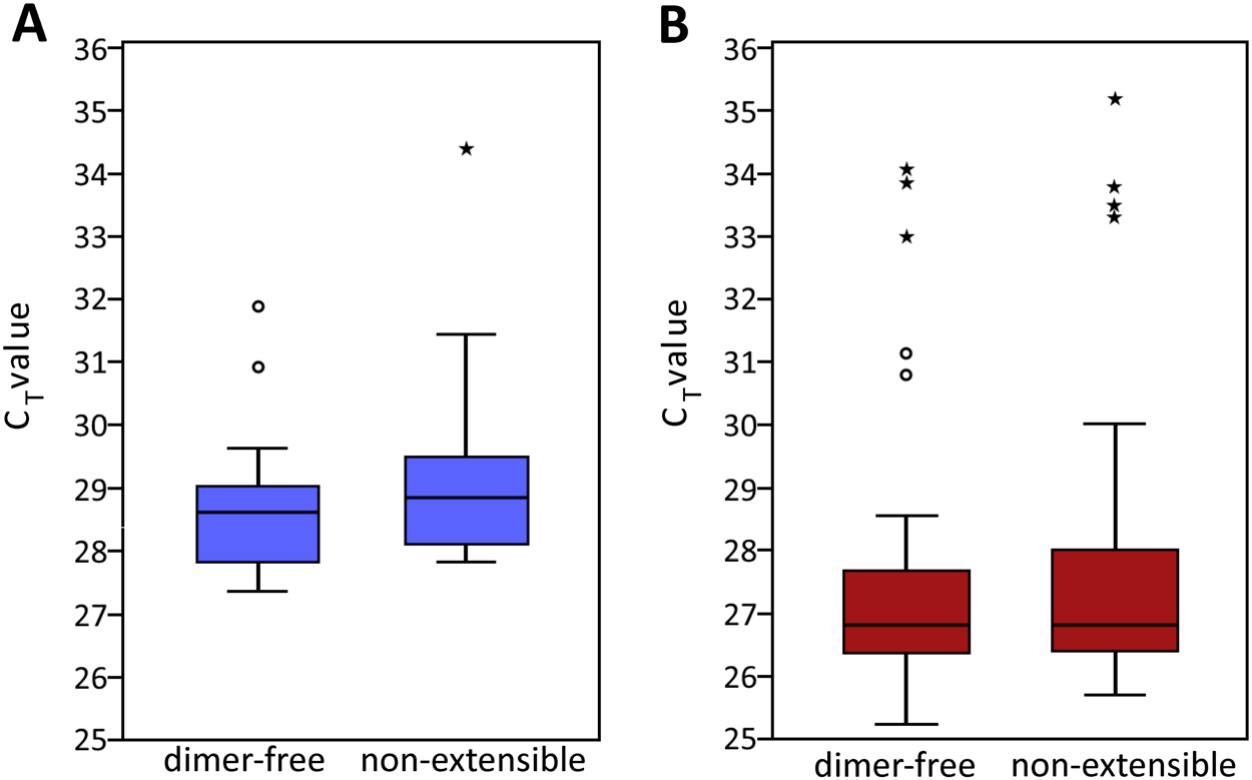
Effect of non-extension dimers on amplification efficiency. A unique genomic region has the same copy number as other unique genomic regions within the same genomic sample, except in rare cases of duplication. Any variation in C_T_ values between primer pairs is thus likely caused by differences in amplification efficiencies. (**A**) 14 base pair fusion primers. No statistical difference between the mean C_T_ values of dimer-free (28.77 ± 1.26, n = 15, 2 outliers) and non-extensible (29.32 ± 1.78, n = 15, 1 outlier) primer pairs (p = 0.250). (**B**) 2 base pair fusion primers. No statistical difference between the mean C_T_ values of dimer-free (27.36 ± 1.87, n = 55, 5 outliers) and non-extensible (27.94 ± 3.70, n = 43, 4 outliers) primer pairs (p = 0.634). The bottom line of each box represents the 25th percentile, top line the 75th percentile, and thick middle line the median. Whiskers extend up to a maximum of 1.5 times the height of the box. Any values that fall outside this range are classified as outliers (circles). Values that are greater than 3 times the height of the box are classified as extreme outliers (asterisks). Statistical analysis performed with IBM SPSS version 24 using Mann-Whitney U Test. ± denotes standard deviation.

Importantly, PrimerDimer does not consider non-extensible dimers in its predictive model. Non-extensible dimers are primer-primer interactions which form stable structures but do not produce spurious dimer-products that elongate and amplify. Non-extensible primer-dimers are easily distinguished from extensible dimers in gel electrophoresis as they tend to be small and faint, and most notably appear as bands in polymerase-free control wells. These bands are generally indistinguishable from primer bands resulting from gel stains with high sensitivity for single-stranded DNA, such as GelRed™. However, staining with ethidium bromide, which has a low sensitivity for single-stranded DNA, will often result in gels that lack the ubiquitous primer banding of GelRed™. When primer-sized bands appear in these gels, they are likely the result of double-stranded non-extensible primer-dimer structures (Supplementary Fig. S6). Considering these structures do not extend and amplify during PCR they are less inhibitory to target amplification, as they do not cause a reduction in available PCR reagents and the primer sequence remains unaltered. To support this hypothesis we performed real-time PCR analysis on two sets of assays—the 2 and 14 base pair fusion primers—which were classified as dimer-free or producing non-extensible dimers, as assessed by PCR and gel electrophoresis. In comparing these two datasets no significant difference was found between the average threshold cycle (C_T_) values of dimer-free versus non-extensible dimer forming primer pairs (Fig. 4). Based on these results, as well as the non-binary and less objective nature of classifying non-extensible dimers (due to their ability to form outside of PCR, their appearance in polymerase-free control wells, and the cross-reactivity of gel stains with single-stranded DNA) PrimerDimer was therefore optimized to predict extensible dimers and exclude non-extensible dimers from consideration. The ability of other dimer tools to predict dimer formation was also assessed on these criteria. In fact, dimer prediction programs often explicitly consider the ability of primer-primer interactions to extend by reporting the most stable 3′-dimer structures.

### Performance comparison of dimer prediction tools using ROC analysis

**Figure 5:**
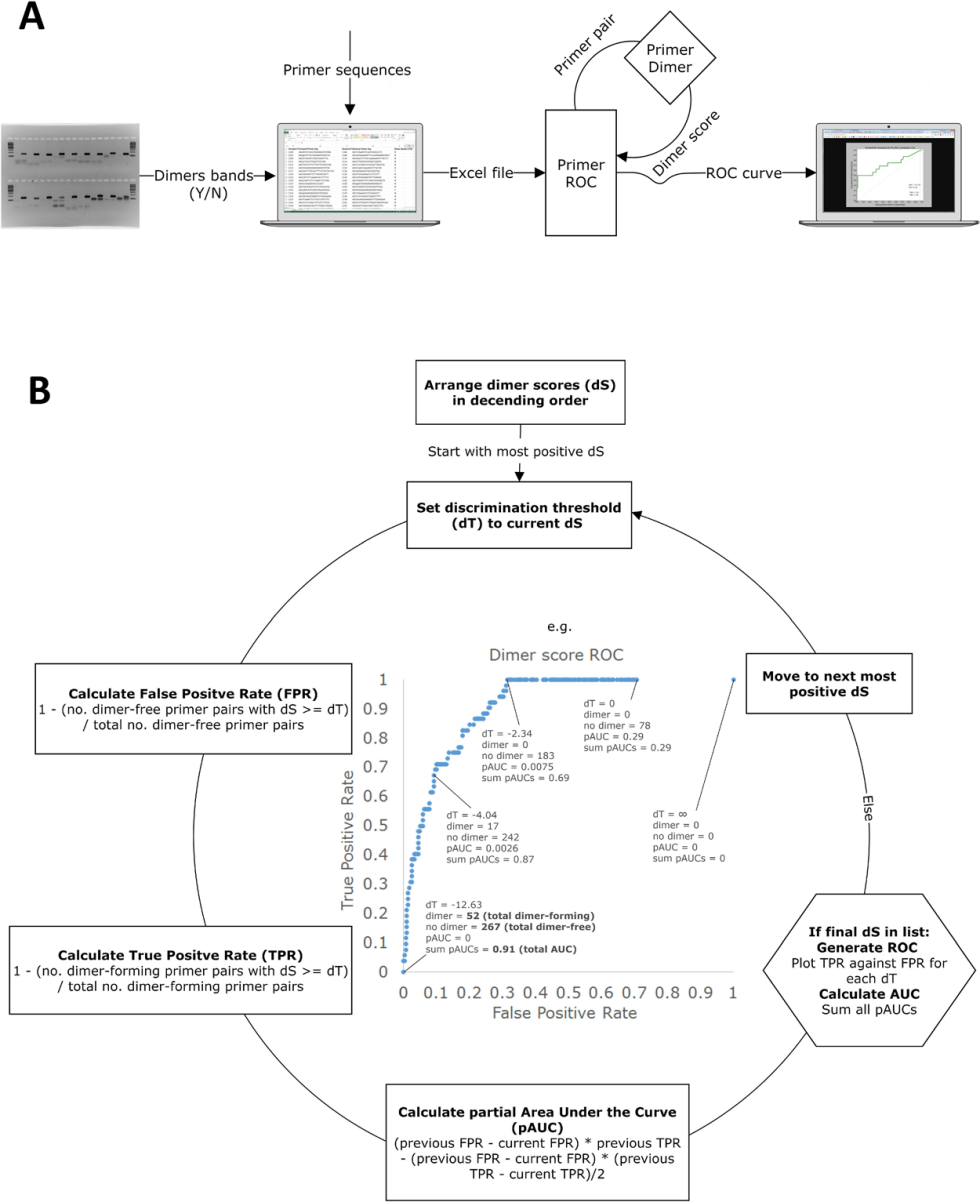
Flow charts detailing how PrimerROC functions. (**A**) User inputs into excel spreadsheet the forward and reverse primer sequences, along with whether or not primer pairs form visible extensible-dimer artefacts in gel electrophoresis. This spreadsheet is uploaded and read by PrimerROC, which then calls the PrimerDimer script to calculate a dimer score for each primer pair. (**B**) A list of all dimer scores paired with dimer band status is arranged from most positive to most negative dimer score. Each dimer score is then sequentially set as the discrimination threshold. The false positive rate (FPR), true positive rate (TPR), and partial area under the curve (AUC) are calculated for each discrimination threshold. These values are then used to plot the receiver operating characteristic (ROC) curve, as seen in the centre example, and calculate the full AUC.

**Figure 6:**
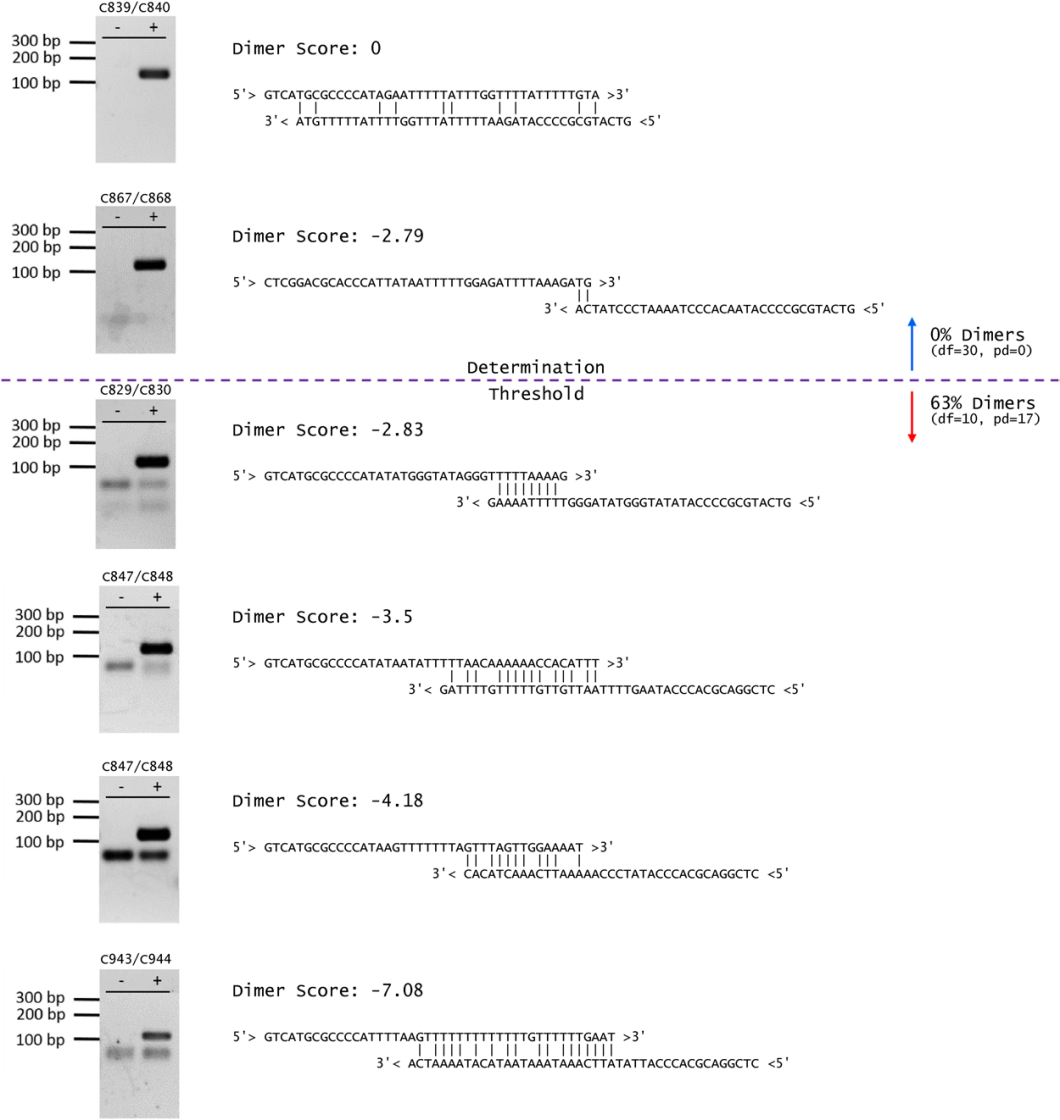
Representative gel electrophoresis images of dimer-forming and dimer-free primer pairs from 14 base pair fusion set. Gel images are accompanied by the predicted dimer structure with the most negative ΔG-based dimer score. The discrimination threshold—score below which primer pairs are classified as dimer forming and above or equal to which primer pairs are classified as dimer-free—is set to the point below which the first dimer forms (dimer score = −2.79). Designing primer pairs above this threshold value allows a user to reliably design dimer-free primer pairs for the same experimental conditions. Gel images were cropped from full gels in Supplementary Fig. S5.

PrimerROC generates ROC curves for sets of primer pairs using PrimerDimer’s ΔG-based dimer scores as the diagnostic maker values and presence or absence of primer-primer PCR amplification artefacts in gel electrophoresis as the dichotomous gold-standard outcomes (Fig. 5). As primer design often requires strict parameters within a highly specific region of DNA, a dimer prediction algorithm should not only provide a high overall accuracy, but also a discrimination threshold where a high percentage of dimer-free primers are correctly classified and few (ideally zero) false negatives occur. As the aim of primer prediction should be to allow users to reliably avoid designing dimer-forming primers pairs, PrimerROC reports the discrimination threshold below which the first dimer forms (Fig. 6). At this cut-off the true positive rate is 1, the false negative rate is 0, and the correct classification of dimer-free primer pairs is maximized.

**Figure 7:**
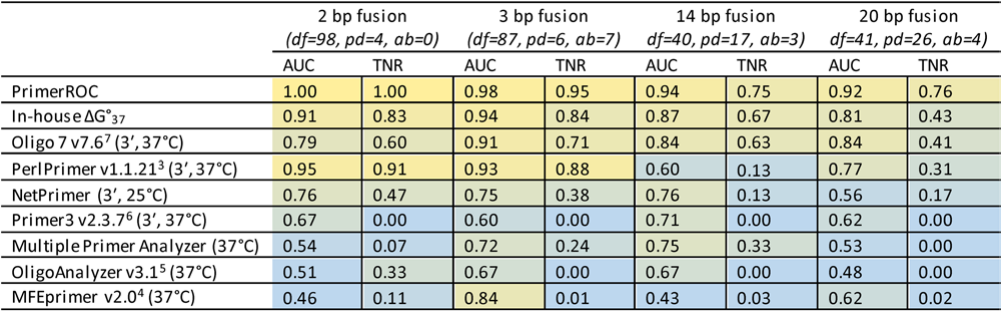
ROC analyses showing performance of dimer algorithms and proportion of correctly identified dimer-free primers. All ΔGs were calculated with standard 1 M Na+, except MFEprimer where the PCR conditions of 50 mM Na+ and 4.5 mM Mg2+ were used. The area under the curve (AUC) has a maximum value of 1 (yellow), with 0.5 being a prediction accuracy no better than chance (blue). The true negative rate (TNR) is at the dimer-free discrimination threshold and has a maximum value of 1 (yellow) and minimum value of 0 (blue). PrimerROC out-performs all other algorithms in every primer set—both in AUC and proportion of correctly classified primers at the dimer-free discrimination threshold. df = dimer free, pd = primer dimer, ab = ambiguous.

Within this framework, PrimerROC consistently out-performed all seven dimer tools in accuracy and dimer-free classification across all four primer sets (Fig. 7 and Supplementary Fig. S7). The only other tool that reliably provided a discrimination threshold above which a substantial proportion of primer pairs were correctly classified as dimer-free was Oligo 7, which performed well across all four datasets and was comparable to our in-house ΔG calculations. The rest of the primer-dimer algorithms we evaluated resulted in dimer-free thresholds with low true negative rates in at least one of the primer sets analysed. While PerlPrimer out-performed Oligo 7 both in overall accuracy and dimer-free classification in the shorter 2 and 3 base pair fusion sets, Oligo 7 considerably out-performed PerlPrimer in the longer sets, particularly the 14 base pair fusion set. This is likely due PerlPrimer only classifying “most stable 3′ extensible primer-dimers” as those with stable structures at both 3′ ends. In our analysis, the primer pairs that formed dimers tended to have strong stable structures at both 3′ ends in the shorter fusion sets, but this was not true of the longer fusion sets.

In all sets, PrimerROC produced an AUC greater than 0.9, an accuracy only achieved by two other tools—PerlPrimer and Oligo 7—and only in the short fusion sets. PrimerROC was able to achieve perfect separation or almost perfect separation of dimer-forming from dimer-free primer pairs in the 2 base pair and 3 base pair fusion sets, respectively. In the 14 base pair and 20 base pair fusion sets PrimerROC correctly classified 75% or greater of the dimer-free primer pairs. Notably, the longer 14 and 20 base pair fusion sets formed dimers much more readily, with dimers forming in scores lower than −2.79 and −2.34, respectively, compared to −6.01 and −6.74 for the 2 and 3 base pair sets. When combining primers from all sets PrimerROC produced an AUC of 0.91 and true negative rate of 0.69 at the dimer-free threshold. However, applying a primer length and G/C percentage adjustment to the primer score increased these values to 0.96 and 0.85, respectively. PrimerROC was thus updated to use this length/GC adjusted dimer score, which provides a dimer-free cut-off of −4.43 (when using our PCR conditions) that is independent of primer length and GC content (Supplementary Data 1).

### Performance of PrimerROC dimer prediction under varying PCR conditions

The PrimerROC method is condition-independent, in that the only input required from the user is their primer pair sequences and dimer-forming status. This allows users to not only determine a dimer-free threshold under varying conditions but also to assess how well the PrimerDimer algorithm performs under the specific conditions used. Dimer prediction algorithms cannot perform equally well under all conditions, as there are conditions that cannot be adjusted for in the calculation of ΔG. The nearest-neighbour thermodynamic parameters used by dimer prediction tools were determined under the specific buffer conditions of 1.0 M NaCl, 10 mM sodium cacodylate, and 0.5 mM NA_2_EDTA, and pH 7.0^11–17^. ΔG calculations can be adjusted for temperature, as well as total sodium concentration^18^. Sodium correction equations can be used to account for all monovalent cations, and the concentration of Mg^2+^ can be converted into a sodium equivalent concentration (such as in NetPrimer). However, other conditions cannot be adjusted for, such as pH or the addition of PCR enhancers (e.g. TMAC, CES, and formamide).

**Figure 8:**
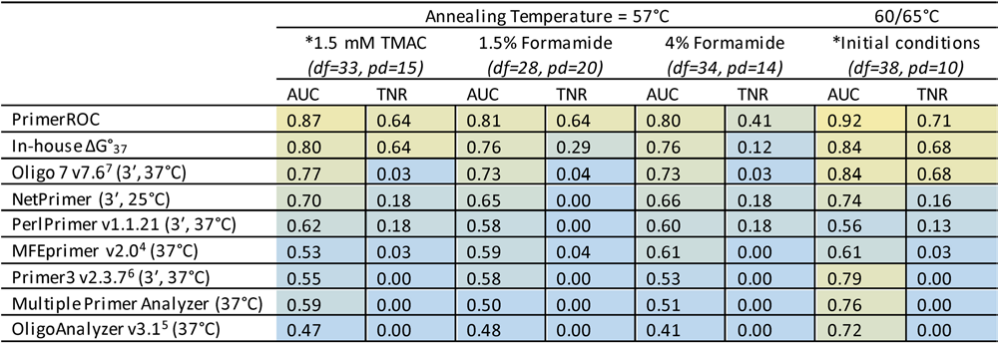
ROC analyses showing dimer prediction performance under altered PCR conditions in 14 bp fusion set. All ΔGs were calculated with standard 1 M Na+, except MFEprimer where the PCR conditions of 50 mM Na+ and 4.5 mM Mg2+ were used. The area under the curve (AUC) has a maximum value of 1 (yellow), with 0.5 being a prediction accuracy no better than chance (blue). The true negative rate (TNR) is at the dimer-free discrimination threshold and has a maximum value of 1 (yellow) and minimum value of 0 (blue). PrimerROC out-performs all other algorithms under each condition tested—both in AUC and proportion of correctly classified primers at the dimer-free discrimination threshold. The predictive performance of PimerROC is also more robust than the other tools under the varying conditions tested. df = dimer free, pd = primer dimer (using stringent dimer classification). Conditions marked with an asterisk (*) contain 0.5X CES. These analyses were performed on the first 48 primers pairs of the 14 base pair fusion set.

Initial evaluation of dimer formation was conducted under similar PCR conditions as those used for resequencing (50 mM sodium, 4.5 mM magnesium chloride, and 0.5X CES). We used an annealing temperature of 57 °C in the shorter 2 and 3 base pair fusion sets, and 60 °C annealing/extension for the first 15 cycles of the 14 and 20 base pair fusion sets. The remaining 20 cycles were run at 65 °C, as fusion sequences do not bind to genomic/bisulfite DNA at the primer’s target site but do bind to subsequent amplicons after the initial PCR cycle. To test the ability of PrimerROC to accurately predict dimers under varying PCR conditions we ran the first 48 primer pairs of the 14 base pair fusion set with a lower 57 °C annealing temperature and added the PCR enhancers tetramethylammonium chloride (TMAC, 1.5 mM; 4 mM MgCl_2_) or formamide (1.5% and 4%; 6 mM MgCl_2_). The overall number of dimers formed, as well as which specific primer pairs formed dimers, varied under each condition (Supplementary Table 2 and Fig. S8). For all predictive performance analyses discussed previously, we classified bands that appeared in the template-free, but not the template lane, as ambiguous and excluded these primer pairs from the analysis. In this case, we were more stringent and classified all primer pairs with template-free bands as dimer-forming, even in the absence of a matching band in the template lane. Under previous conditions, 8 of these 48 primer pairs were classified as dimer-forming and 2 were classified as ambiguous (here reclassified as dimer-forming). These new conditions all resulted in dimers forming more readily, with 15 classified as dimer-forming in the 1.5 mM TMAC; 20 in the 1.5% formamide; and 14 in the 4% formamide. PrimerROC, this time using the length/GC adjusted dimer score, again out-performed all other tools in overall accuracy and dimer-free classification. In addition, the performance of these other tools, particularly their ability to produce dimer-free thresholds, was diminished under these conditions. This was also true of our own ΔG calculations in the case of formamide. However, due to its inclusion of bonuses and penalties that are independent of ΔG, PrimerROC was buffered against these changes and experienced a considerably reduced negative impact on performance (Fig. 8 and Supplementary Fig. S9).

### PrimerROC compared to temperature and salt adjusted ΔG

**Figure 9:**
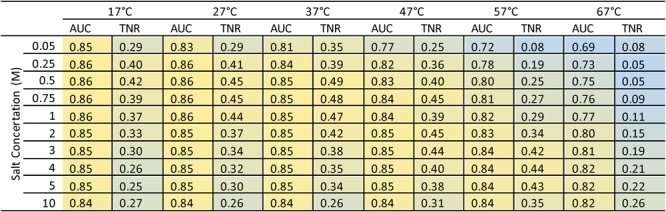
ROC analyses showing dimer prediction of in-house ΔG calculated with varying temperatures and salt concentrations. The area under the curve (AUC) has a maximum value of 1 (yellow = 0.87), with 0.5 being a prediction accuracy no better than chance (blue = 0.69). The true negative rate (TNR) is at the dimer-free discrimination threshold and has a maximum value of 1 (yellow = 0.5) and minimum value of 0 (blue). Colours correspond to the maximum and minimum values within this set, allowing for better visualization of differences between conditions. Altering temperature and sodium concentration parameters when calculating ΔG can greatly impact its predictive accuracy. However, the standard 37 °C with 1 M sodium approaches maximum accuracy, with a lower sodium concentration of 0.5 M giving slightly better performance. PrimerROC results in substantially greater prediction accuracy over ΔG alone (AUC = 0.94 and TNR = 0.56). Analyses were performed on the combined set of all fusion primers using stringent dimer classification.

It is possible that the dimer prediction performance of ΔG alone might be considerably improved by adjusting temperature and sodium concertation values in its calculation. We, therefore, tested temperatures ranging from 17–67 °C and sodium concentrations between 0.05–10 M to look for peaks in performance when using our in-house ΔG to predict dimer formation. These tests were performed using the combined set of all fusion primers with stringent dimer classification, where all bands originally classified as ambiguous were reclassified as dimer-forming. Increasing temperature from the standard 37 °C actually reduced predictive performance, whereas decreasing temperature resulted in a mild increase. Predictive performance was found to depend on the combination of temperature and sodium concentration, with performance for lower temperatures peaking at lower sodium concentrations, and vice versa. The overall peak in predictive performance for all combinations was 37 °C at 0.5 M sodium, which produced an AUC of 0.85 and true negative rate of 0.49, compared to PrimerROC’s 0.94 and 0.56. We also performed these same analyses for each fusion primer set separately using stringent and non-stringent dimer classification and found similar results (data not shown). Notably, changing the temperature and salt conditions when calculating ΔG to better reflect those of the PCR conditions used had a substantially negative impact on predictive performance compared to the standard ΔG°_37_ in 1 M sodium (Fig. 9 and Supplementary Fig. S10).

**Figure 10:**
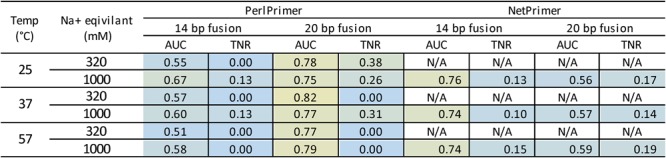
ROC analyses of dimer prediction tools using varying temperatures and salt concentrations to calculate ΔG. PerlPrimer and NetPrimer both allow users to change the temperature at which ΔG is calculated. PerlPrimer also allows user to alter cation concentrations that affect the calculation of ΔG. Three temperatures were tested with standard 1 M Na+, as well as the cation concentrations 50 mM Na+ and 4.5 mM used in PCR (equivalent to 320 mM Na+ when calculating ΔG). The area under the curve (AUC) has a maximum value of 1 (yellow), with 0.5 being a prediction accuracy no better than chance (blue). The true negative rate (TNR) is at the dimer-free discrimination threshold and has a maximum value of 1 (yellow) and minimum value of 0 (blue). Analyses were performed on the longer 14 and 20 base pair fusion primer sets.

Most of the tools tested use the standard 37 °C in 1 M sodium to calculate ΔG. However, NetPrimer and PerlPrimer allow users to input the temperature at which ΔG is calculated. We, therefore, tested these tools at their default 25 °C, the standard 37 °C, and a high 57 °C when determining the ΔG for the longer 14 and 20 base pair fusion sets to see how this would impact their predictive performance. Overall, the effects of temperature were minor in both tools, although PerlPrimer was better able to produce a dimer-free threshold at the lower 25 °C. Additionally, the input of cation concentrations also influence the calculation of ΔG in PerlPrimer. We, therefore, tested the standard 1 M sodium as well as the sodium and magnesium concentrations used in our PCR runs (equivalent to 0.32 M sodium) to see how cation concentration altered predictive performance. Performance peaked at 25 °C at 1 M for the 14 base pair set and 25 °C at 320 M for the 20 base pair set. The combined effects of temperature and cation concentration were minor on AUC, but did influence the true negative rate and whether or not a dimer-free threshold was established (Fig. 10).

### Dimer-free multiplex resequencing assays

**Figure 11:**
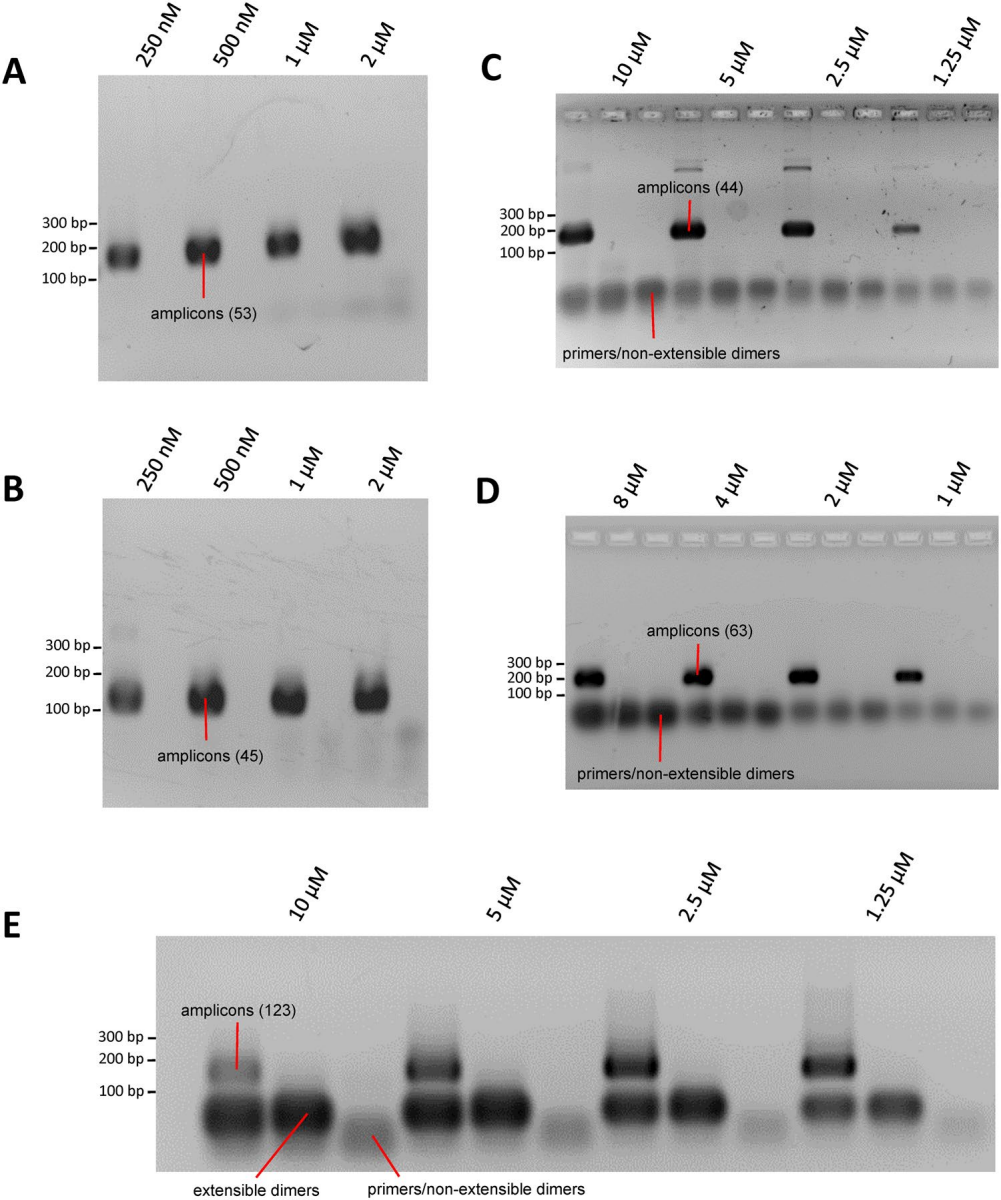
Multiplex PCR of four dimer-free and one primer-forming resequencing assays at various total primer concentrations. The first lane for each concentration contains template, the second is template-free. The third lane for each concentration in (**C**–**E**) is template-free and polymerase-free. (**A**) Neurological bisulfite PCR pool 1 (53 primer pairs). (**B**) Neurological bisulfite PCR pool 2 (45 primer pairs). (**C**) Somatic mutations in cancer panel genomic PCR (44 primer pairs). (**D**) SNPs in sugarcane panel genomic PCR (63 primer pairs). (**E**) Example of a multiplex bisulfite assay (123 primer pairs) where PrimerROC was not used and visible dimers formed under the same PCR conditions as the dimer-free assays in **A**-**D**. Gel images were cropped from full gels in Supplementary Fig. S11.

One application of accurate dimer prediction is the possibility to design large multiplex reactions which concurrently amplify multiple targets but are devoid of dimer artefacts. To demonstrate the feasibility of this approach we incorporated PrimerDimer and our PrimerROC derived dimer-free threshold into an in-house version of our primer design tool PrimerSuite^19^, and combined this with a modified version of our multiplexing tool PrimerPlex, to produce a multiplex design procedure that minimizes the likelihood of primer-primer elongation for each new region added to the growing multiplex pool. Using this procedure we produced four PCR/resequencing multiplex reactions each with between 43–63 primer pairs with no visible dimer products (Fig. 11A–D). Multiplexes with this many primers generally result in a high density of extensible dimer artefacts when designed with poor dimer prediction accuracy, particularly at overall primer concentrations of up to 10 µM. An example of such dimer formation can be seen in the template and no template lanes (but not in the polymerase free lane) of a multiplex we designed previously without using PrimerDimer/PrimerROC (Fig. 11E). Increasing primer concentration in this dimer-forming multiplex appears to diminish overall product yield, as more primers accelerate dimer formation, which then outcompetes binding to target sites.

**Figure 12:**
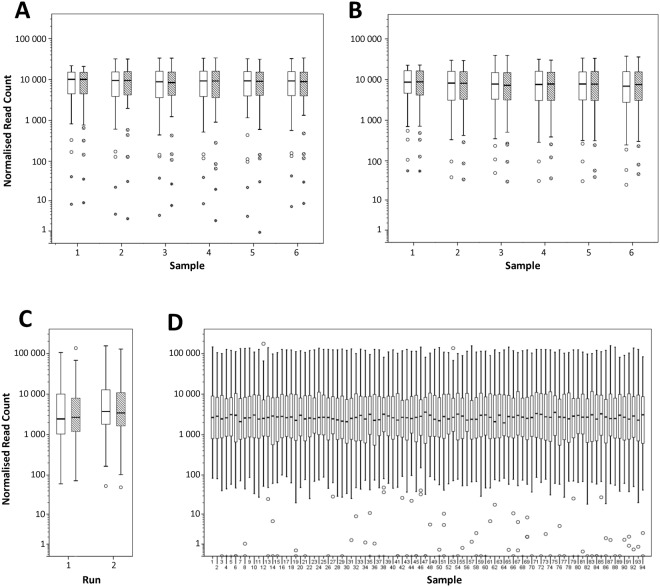
Box and whisker plots depicting the read count distributions for primer pairs within each multiplex resequencing assay. Each pool is normalized to have an average of 10,000 reads per primer pair. Read counts vary between primer pairs due to differences in primer efficiencies and the multitude of thermodynamically driven binding interactions competing in a multiplex primer pool. These graphs represent the read count spread of primer pairs within each of the four multiplex primer pools tested. (**A**) Neurological bisulfite resequencing pool 1 (**B**) Neurological bisulfite resequencing pool 2. **A**,**B** were run with two technical replicates (solid white and black stripes) on six samples of varying methylation levels. (**C**) Somatic mutations in cancer genomic resequencing panel showing technical replicates on a single blood-pool sample across two independent sequencing runs. (**D**) SNPs in sugarcane genomic resequencing panel run on 94 sugarcane samples. The bottom line of the box represents the 25th percentile, top line the 75th percentile, and thick middle line the median. Whiskers extend up to a maximum of 1.5 times the height of the box. Any values that fall outside this range are classified as outliers (circles). Values that are greater than 3 times the height of the box are classified as extreme outliers (asterisks).

Primers within these multiplex pools were designed to amplify bisulfite converted regions associated with neurological disease for CpG methylation detection, genomic regions associated with high levels of somatic cancer mutations, and single nucleotide polymorphisms in sugarcane (Fig. 12). All four multiplexes performed well, with >96 million reads per run and a throughput of >14.2 Gb (system specifications of 60–80 million and up to 15 Gb, respectively). Dropouts, where a primer pair produced few or no reads within a multiplex pool, were rare and read counts for each primer pair within a multiplex pool spanned ~4 orders of magnitude. Amplification of multiplex pools was performed with total primer concentrations of 2 µM (15.9–23.3 nM per primer). We have since observed in real-time PCR experiments that differences in primer efficiencies/product yield between primer pairs greatly increases at low primer concentrations (<40 nM per primer) and that increasing to a total primer concentration of 10 µM in multiplex resequencing (a concentration at which we still observe no dimer artefacts) substantially reduces the variability in read count between multiplexed primer pairs (data not shown). PrimerROC thus provides a powerful tool for assay design, as it allows users to effectively prevent dimers in reactions and at primer concentrations where they have historically been difficult to avoid.

## Material and Methods

### Assessment of primer-dimer prediction programs

To determine the accuracy of different dimer algorithms in predicting the formation of primer-dimers, the dimer score (i.e. the value used by each program to report dimers) for the most stable structure of each primer pair was entered into a spreadsheet along with the forward and reverse primer sequences and whether dimer artefacts were observed in gel electrophoresis of both template and template-free PCR runs. Bands where it was unclear whether they were the result of extensible or non-extensible dimers, including artefact bands that were extremely faint in both template and template-free lanes, appeared in only the template-free but not the template lane, or were very small but dark when compared to amplicon bands, were excluded from analysis. Due to the high number of ambiguous bands that appeared on the first run of the 20 base pair fusion primer set, PCR and gel electrophoresis was performed an additional two times in order to resolve these ambiguities. Primer tables were then loaded into an in-house ROC generating python script to create ROC curves reporting the AUC and dimer-free discrimination threshold. Where programs reported both most stable overall and 3′-dimer structures, we assessed the ability of both these scores to predict dimer formation and reported results of the method with greater performance.

#### PerlPrimer

Used PerlPrimer version 1.1.21 to assess dimer prediction. The PCR component concentrations under the General tab were set to the conditions used in the Bisulfite PCR Conditions section below (Mg++: 4.5 mM; Oligos: 0.25 µM; dNTPs: 0.05 mM; Monovalent cations: 50 mM). Forward and reverse primer sequences were input under the Primers tab to calculate the ΔGs of the most stable homodimer and heterodimer structures. The temperature for ΔG calculation was set to 37 °C under Preferences > Dimers to standardize with other programs that offer a static 37 °C without the option to alter the temperature parameter. In addition, ΔGs were also calculated at PerlPrimer’s default 25 °C, as well as 57 °C, for the 14 and 20 base pair fusion sets to see if changing the temperature parameter to calculate ΔG increased predictive power. Each temperature was also paired with the cation conditions listed above (equivalent to 320 mM of monovalent cations), as well as 1000 mM monovalent cations (0 mM Mg++) ROC curves were generated for both the most stable non-extensible primer-dimers and most stable 3′ extensible primer-dimers values using the most negative ΔG for each primer pair.

#### Oligo 7

Used Oligo 7 version 7.60 (DEMO) to assess dimer prediction. Forward Primer and Reverse Primer sequences were input under the Edit menu and the ΔGs of the most stable overall and 3′-dimer of the Forward Primer, Reverse Primer, and Mixed Oligos were recorded from Analyze > Duplex Formation. ROC curves were generated for both the overall and 3′-dimer values using the most negative ΔG for each primer pair. Oligo 7 does not offer the options to alter temperature or cation concentration in the calculation of ΔG.

#### OligoAnalyzer

Used OligoAnalyzer version 3.1 to assess dimer prediction. The PCR sets were set to the conditions used in the Bisulfite PCR Conditions section below (Target type: DNA; Oligio Conc: 0.25 µM; Na+ Conc: 50 mM; Mg++ Conc: 4.5 mM; dNTPs Conc: 0.05 mM). However, these parameters do not appear to influence the resulting ΔG values and are only used in the calculation of melting temperature. Forward and reverse primer sequences were input separately to determine the ΔGs of the most stable homodimers using the Self-Dimer function. The most stable heterodimer structure was then determined using the Hetero-Dimer function. OligoAnalyzer does not offer the option to alter temperature.

#### MFEprimer

Used MFEprimer version 2.0 to assess dimer prediction. The Experimental settings were set to the conditions used in the Bisulfite PCR Conditions section below (Annealing oligo concentration: 0.25 µM; Concentration of monovalent cations: 50 mM; Concentration of divalent cations: 4.5 mM; Concentration of dNTPs: 0.05 mM). Forward and reverse primer sequences were input to determine the ΔGs of the most stable homodimer and heterodimer structures. MFEprimer 2.0 does not offer the option to alter temperature.

#### NetPrimer

Used NetPrimer to assess dimer prediction between 24 March 2016 and 22 April 2016. The Reaction Conditions were set to the conditions used in the Bisulfite PCR Conditions section below (Oligo Concentration: 0.25 µM; Monovalent Ion Concentration: 50 mM; Free Mg++ Ion Concentration: 4.5 mM). However, these parameters do not appear to influence the resulting ΔG values and are only used in the calculation of melting temperature. Forward and reverse primer sequences were input to determine the ΔGs of the most stable Self Dimer (homodimer) and Cross Dimer (heterodimer) structures. The Temperature for Free Energy Calculation was set to 37 °C to standardize with other programs that offer a static 37 °C without the option to alter the temperature parameter. In addition, ΔGs were also calculated at NetPrimer’s default 25 °C, as well as 57 °C, for the 14 and 20 base pair fusion sets to see if this increased predictive power. ROC curves were generated using both the minimum (most negative) ΔG and minimum 3′ Dimer ΔG for each primer pair.

#### Multiple Primer Analyzer

Used Multiple Primer Analyzer to assess dimer prediction between 24 March 2016 and 30 May 2016. The Parameters for calculation of primer Tm were set to the conditions used in the Bisulfite PCR Conditions section below (Primer concentration: 0.25 µM; Salt concentration: 50 mM). However, these parameters do not influence the presence of dimers at different sensitivity settings and are only used in the calculation of melting temperature. Forward and reverse primer sequences were input in FASTA format and the Value of the sensitivity for dimmer detection was sequentially raised from the maximum sensitivity (1) to the minimum sensitivity (10). The sensitivity before which dimer structures were no longer present in the ‘Results for primer-dimer detection’ output box was then recorded for each primer pair. Less stable structures require more sensitivity to detect, meaning the most stable structures remain the longest as the sensitivity is lowered. If no dimers were present at the maximum sensitivity of 1 the value recorded was 0. Multiple Primer Analyzer does not offer the option to alter temperature.

#### Primer3

Used Primer3 version 2.3.7 to assess dimer prediction. Primer3 was installed on our Linux server via the instructions presented in the Primer3 Release 2.3.7 Manual. Forward and reverse sequences were pasted into the inputfile.txt and primer3_core was then run on this file. The most negative overall ΔGs for homodimers and heterodimers, as well as the most negative 3′ ΔG for homodimers and heterodimers, were then retrieved from the stdout. Primer3 does not offer the options to alter temperature or cation concentration in the calculation of ΔG.

### PrimerROC Implementation

The PrimerROC/PrimerDimer software was originally written in the Python language (version 3.4+) (https://www.python.org), and later adapted into a web application using the Django framework (version 1.8+) (https://www.djangoproject.com), and hosted via Apache2 HTTP server (https://httpd.apache.org) on the nectar cloud (https://nectar.org.au).

### PrimerDimer dimer score calculation

PrimerDimer begins by receiving two oligo sequences in the 5′ to 3′ orientation. Out of these two sequences, the longer oligo is assigned the “top”, while the shorter is assigned the “bottom” and its sequence is reversed into the 3′ to 5′ orientation. The 3′ end of the bottom oligo is then paired to the 5′ end of the top oligo, creating a dimer structure with a single 3′ overhang. The bottom oligo then slides to the right (3′ of the top oligo) to create all possible dimer structures with 5′ overhangs, with each end treated independently.

PrimerDimer starts from the first two bases at the 3′ end and determines their ΔG (kcal/mol) using nearest-neighbour (NN) parameters for duplexes^11^ and single mismatches^12–16^, or assigns a penalty for double mismatches. The NN ΔGs of subsequent nucleotide pairs are then progressively added to determine the dimer structure’s ΔG. The most negative ΔG derived from a single NN iteration of this process is taken as the dimer score. Next, penalties are added for single base mismatches in the first few bases from the 3′ end of the dimer structure, and a further penalty is added if either the first or second base is a mismatch in addition to at least one other mismatch in the first 5 bases. The ΔGs of 5′ overhangs^17^ are then added and finally, a bonus is added if the first base of the dimer structure is a C or G and the structure spans both 3′ ends with 100% of base pairs matched. All bonuses and penalties were derived by iterating through a series of values for each parameter on the 3 and 20 base pair training sets. Those values that gave the maximum dimer-free true negative rate (and, where tied, the maximum AUC) were used in the final algorithm.

The dimer score (dS) length/GC adjustment was also derived from this method. The pseudocode for the calculation of the length/GC adjusted dS is detailed below:

$$\begin{array}{c}{\rm{dS}}=\mathrm{dS}/\mathrm{20}\ast (\mathrm{length}\,{\rm{of}}\,{\rm{top}}\,{\rm{primer}}+{\rm{length}}\,{\rm{of}}\,{\rm{bottom}}\,\mathrm{primer})\,\#\,{\rm{length}}\,{\rm{adjustment}}\\ {\rm{dS}}={\rm{dS}}+{\rm{dS}}\ast ({\rm{average}}\,{\rm{GC}}\,{\rm{count}}\,{\rm{of}}\,{\rm{top}}\,{\rm{and}}\,{\rm{bottom}}\,{\rm{primer}})\ast {\rm{2.25}}\,\#\,{\rm{G}}/{\rm{C}}\,{\rm{adjustment}}\\ {\rm{dS}}={\rm{dS}}/4.4\,\#\,{\rm{adjustment}}\,{\rm{to}}\,{\rm{make}}\,{\rm{more}}\,{\rm{comparable}}\,{\rm{to}}\,{\rm{\Delta }}{\rm{G}}\end{array}$$

### Multiplex design procedure

This procedure iterates through a list of input DNA regions, producing all viable forward and reverse primer pairs. Regions are then sorted from those with the least to those with the most primer pairs. Prospective primers for each region are paired with all other primers within the existing pool and the most negative ΔG-based dimer score of all these interactions is recorded. If the score is within the dimer-free range, the primer pair is stored as a candidate for its particular region. The primer pair with the highest (least negative) dimer score for a region is then added to the growing multiplex pool.

### Bisulfite DNA conversions

Bisulfite conversion of template DNA was conducted using manual protocols reported previously^20^. For each conversion, DNA was first quantified with the Qubit dsDNA BR Assay Kit and, based on the available sample material, between 100 ng – 1 µg of material was bisulfite converted at a time. Conversion took place at 80 °C for 45 minutes, followed by resuspension in low TE (10 mM Tris-CL, pH 8.0, 0.1 mM EDTA).

### Bisulfite PCR conditions for determining the presence of dimer artefacts

A PCR mastermix recipe for amplification of bisulfite-converted DNA was made and the final PCR reaction had the following components at the indicated concentrations: 5X Promega GoTaq; 5X Green Flexi Buffer (1X final, PN M5005); CES 5 × (0.5X final, N.B. refer to Ralser *et al*.^21^ for CES recipe); MgCl_2_ (4.5 mM final); dNTP’s (0.05 mM each final); primers (forward and reverse at 0.25 µM final); Taq (0.025 U/µL final); DNA (variable final concentration, but typically not exceeding 2 ng/µL final concentration). Amplification took place on either an Eppendorf ProS 96 well, or Eppendorf Pro 384 well thermocycler. Cycling conditions were as following: Primers with 2 and 3 base pair fusion sequences: 35 cycles (95 °C for 20 s, 57 °C for 15 s, 72 °C for 45 s); Primers with 14 and 20 base pair fusion sequences: 15 cycles (95 °C for 20 s, 60 °C for 60 s); and 20 cycles (95 °C for 20 s, 65 °C for 90 s). All reactions started with a 10 min 95 °C step for activation of the Hot-Start GoTaq® DNA Polymerase and finished with a 10 °C hold. PCR products were evaluated using standard 2% agarose gel electrophoresis techniques with SB buffer and ethidium bromide. Each primer pair was screened against two samples: a bisulfite–converted DNA template and no template control. The three altered reagent and cycling conditions for the 14 base pair fusion set were the same as those of the 2 and 3 base pairs set conditions detailed above with the following differences: (1) 1.5 mM TMAC, 4 mM MgCl_2_; (2) 1.5% formamide, 6 mM MgCl_2_, no CES; (3) 4% formamide, 6 mM MgCl_2_, no CES.

### Real-time PCR of dimer-free and non-extensible dimer forming primer pairs

Real-time PCR was run on the Applied Biosystems® 7500 Real-Time PCR with the same mastermix recipe and cycling conditions as the bisulfite PCR conditions detailed above, with the addition of SYTO™ 9 Green Fluorescent Nucleic Acid Stain (2 µM final) and ROX reference dye for real-time PCR (0.5 µM final), and replacement of 5X Green Flexi Buffer with 5X Colorless Flexi Buffer. Non-extensible dimers were determined by their appearance as bands in three separate samples on ethidium bromide stained gel electrophoresis. These samples included: a bisulfite–converted DNA template, no template control, and Taq-free control.

### PCR for sequencing conditions

Initial amplification cycling conditions were: 95 °C, 10 mins; 15 cycles of (95 °C, 15 s; 57 °C short or 60 °C long fusion, 30 s; 72 °C, 90 s); 72 °C, 2 mins; 4 °C, 5 mins. The final concentrations for each reaction were: 1x Promega GoTaq Green Flexi buffer; 4.5 mM MgCl_2_; 200 µM dNTPs; 0.05 U/µL HotStart Taq. DNA from each reaction was then purified using Agencourt XP bead cleanup and individually amplified with Ion Xpress™ Barcode Adapters. Cycling conditions were: 95 °C, 10 mins; 15 cycles (95 °C, 20 s; 45 °C, 20 s; 68 °C, 60 s); 68 °C, 2 mins; 4 °C, 5 mins. Barcoded PCR products were then pooled and excess primers were extracted using gel purification (Promega gel cleanup kit). Amplification took place on either an Eppendorf ProS 96 well, or Eppendorf Pro 384 well thermocycler.

### Sequencing of multiplex pools

Sequencing was performed on the Ion Proton™ System. Template preparation was performed with the Ion OneTouch™ 2 System (OT2) using the Ion PI™ Hi-Q OT2 200 Kit. Proton runs used the Ion PI™ Chip Kit v2 and Ion PI™ Hi-Q Sequencing 200 Kit.

### Sequencing of dimer artefacts

Dimer artefacts were derived from no-template controls and barcoding was performed using commercially purchased oligonucleotides with MiSeq sequencing adaptors and barcodes (Fluidigm PN FLD-100-3771). Sequencing was performed on the Illumina Miseq. MiSeq runs used the MiSeq Reagent Kit v2, 300 cycle; PN MS-102-2002.

### Bioinformatics

Adaptor trimming used Trim galore (options:–length 70,–clip_R1 <length of barcode>, -a <forward barcode>). Mapping of sequenced data used Bowtie 2^22^ version 2.3.0-legacy for genomic and Bismark^23^ running Bowtie 2 (options: –bowtie2 –N 1 -L 15 –bam -p 2 –score L, −0.6, −0.6 –non_directional) for bisulfite converted DNA. Graphing and statistical analysis employed IBM SPSS Statistics 24.

## Electronic supplementary material

Supplementary Info

## Data Availability

The PrimerROC tool is available at http://www.primer-dimer.com/roc/ as a web application.
