# Supplementary material for: PrimerROC: accurate condition-independent dimer prediction using ROC analysis
Source: Sci Rep. 2019 Jan 18;9:209. doi: 10.1038/s41598-018-36612-9 (PMC6338771; doi:10.1038/s41598-018-36612-9)
Supplement: Supplementary file 1 — Supplementary Info [file 41598_2018_36612_MOESM1_ESM.zip › 02july2018_SciRep_Supplementary_Figures_Revised.pdf]

Supplementary file

Title: PrimerROC: accurate condition-independent dimer prediction using ROC analysis

Andrew D Johnston, Jennifer Lu, Ke-lin Ru, Darren Korbie, Matt Trau

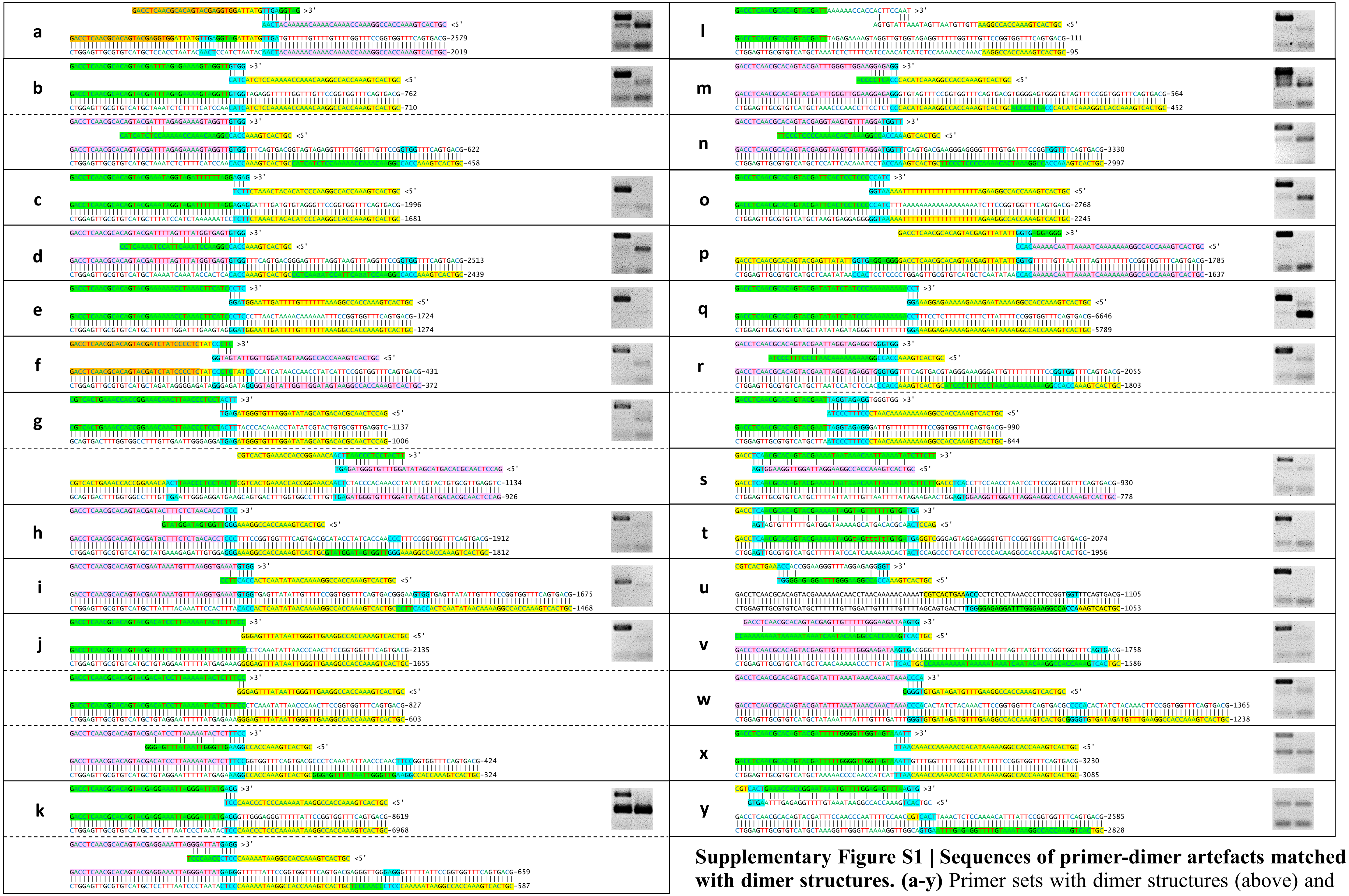

Supplementary Figure S1 | Sequences of primer-dimer artefacts matched with dimer structures. (a-y) Primer sets with dimer structures (above) and artefact sequences arranged in compliments (below). Gel image displayed to right of each primer set. Sequence read count beside each sequence.

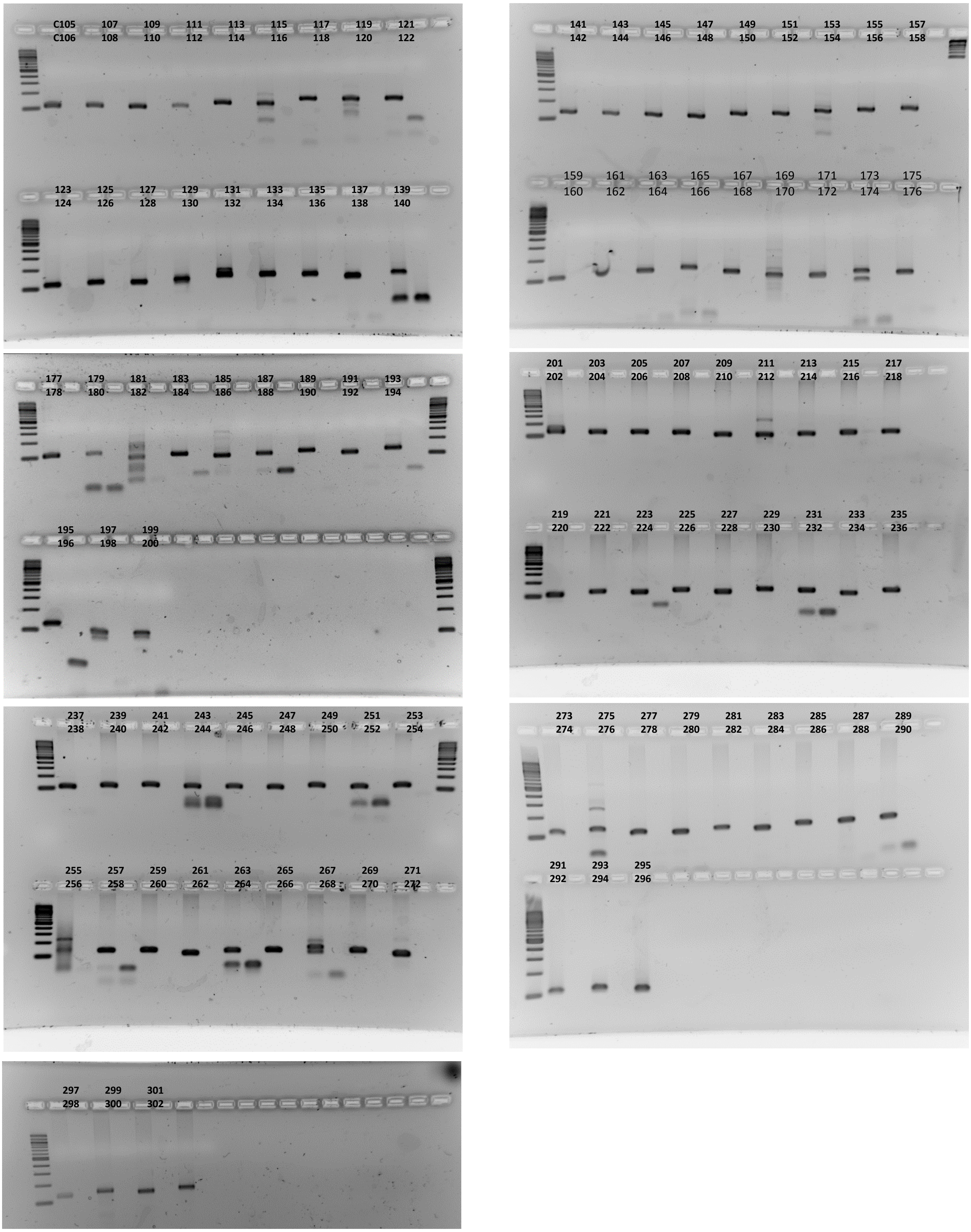

Supplementary Figure S2 | Gel electrophoresis images of 3 base pair fusion primer PCR products and dimer artefacts. First lane of each primer pair contains template and second lane is template free. Ladder is 100 bps. Stained with ethidium bromide.

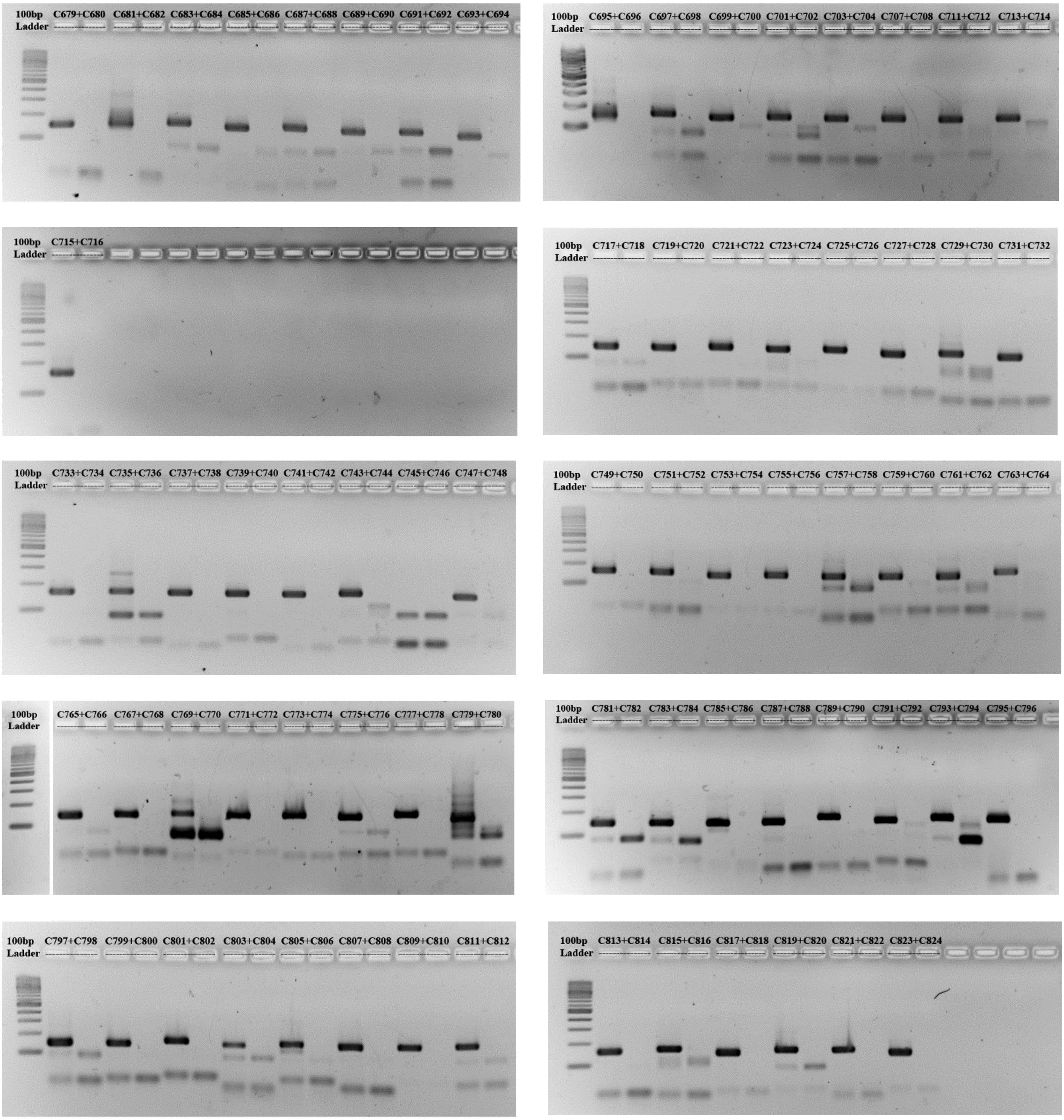

Supplementary Figure S3 | Gel electrophoresis images of 20 base pair fusion primer set PCR products and dimer artefacts. First lane of each primer pair contains template and second lane is template free. Ladder is 100 bps. Stained with ethidium bromide.

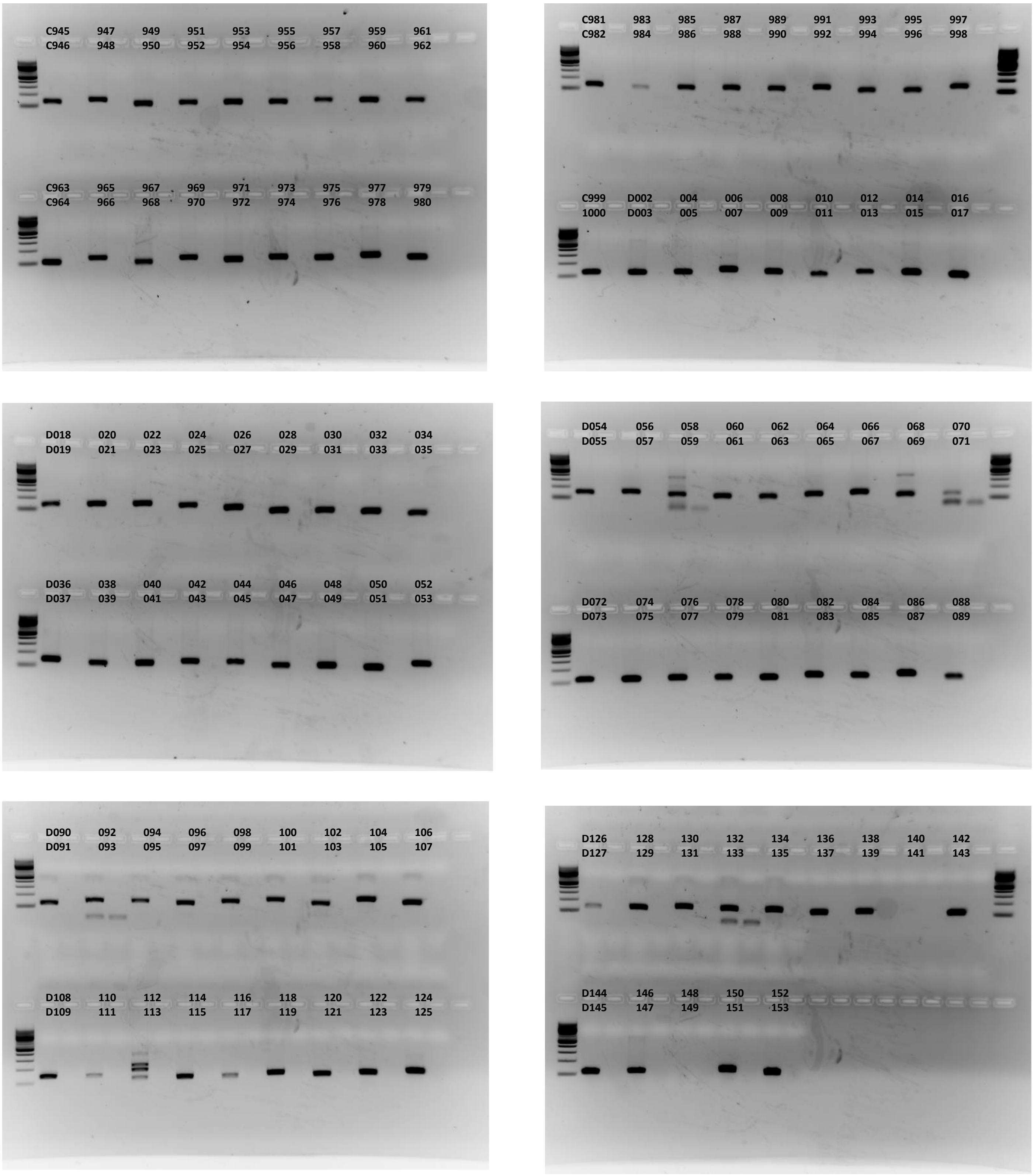

Supplementary Figure S4 | Gel electrophoresis images of 2 base pair fusion primer PCR products and dimer artefacts. First lane of each primer pair contains template and second lane is template free. Ladder is 100 bps. Stained with ethidium bromide.

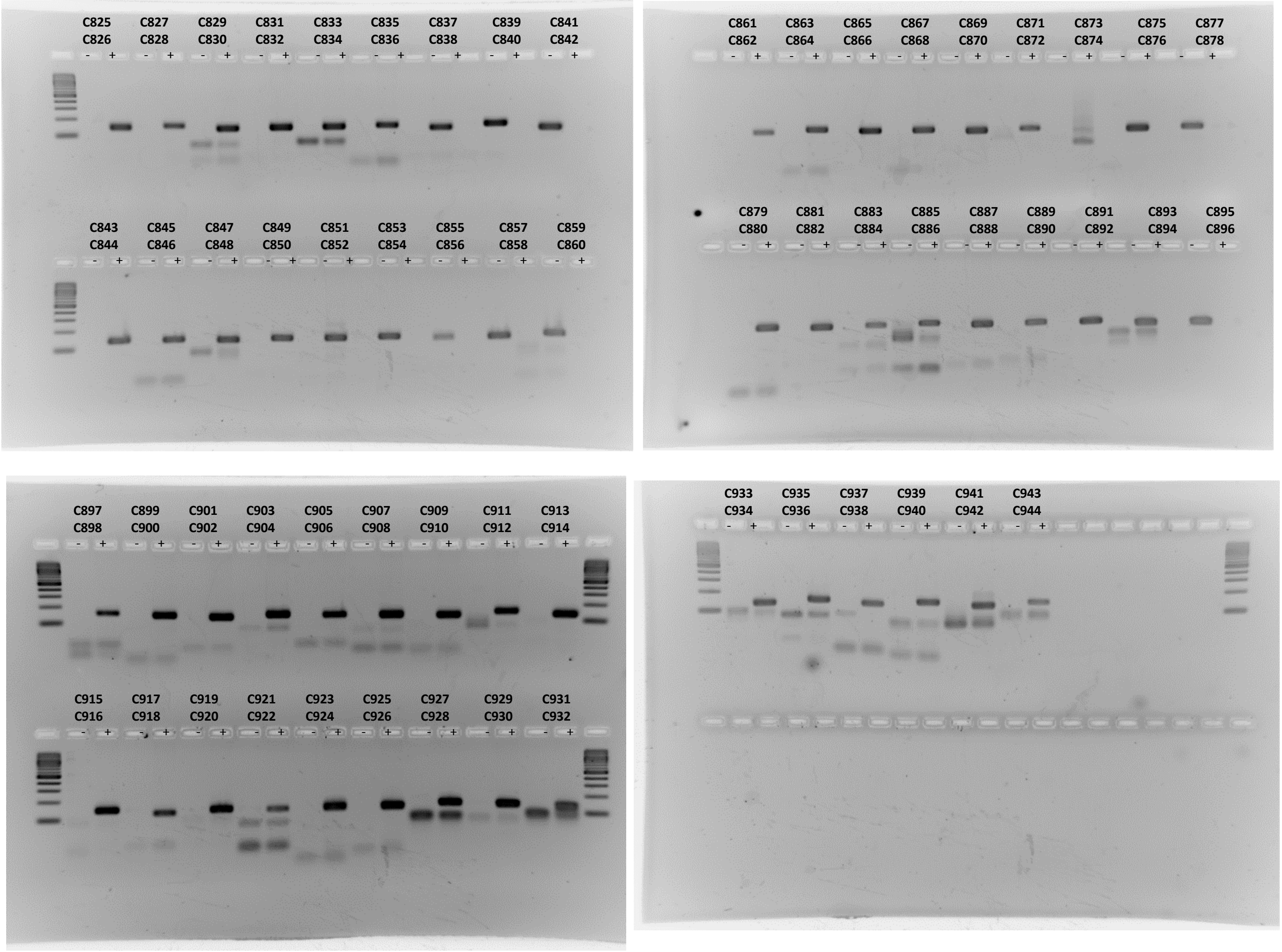

Supplementary Figure S5 | Gel electrophoresis images of 14 base pair fusion primer PCR products and dimer artefacts. Second lane of each primer pair contains template and first lane is template free. Ladder is 100 bps. Stained with ethidium bromide.

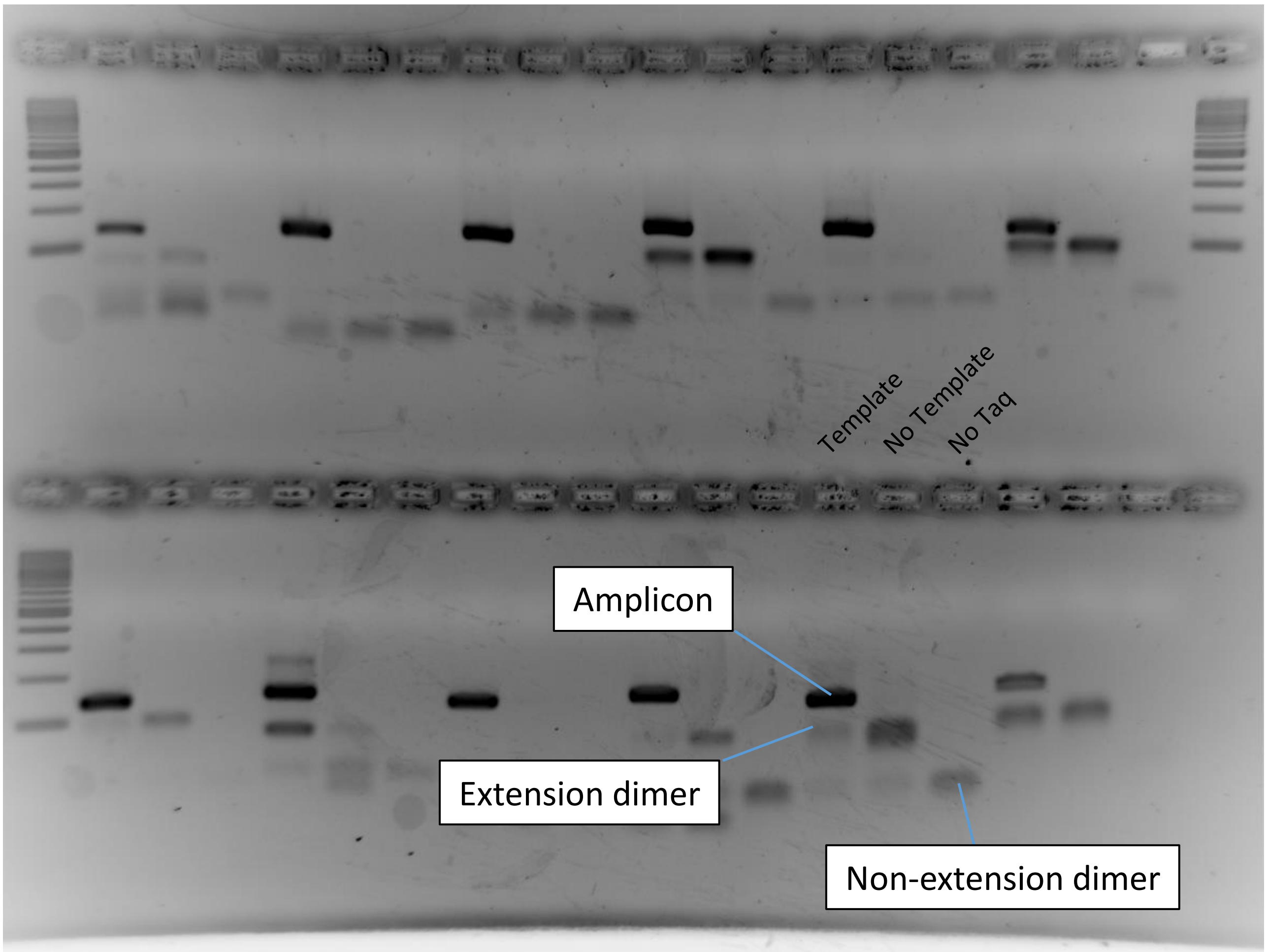

Supplementary Figure S6 | Appearance of non-extension dimers in gel electrophoresis stained with ethidium bromide. Non-extension dimers are distinguished from extension dimers by their small size and appearance as bands in polymerase-free controls. Ethidium bromide binds to DNA helical structures. In the absence of DNA template the only helical structures arising from elongation are those of extension dimers, which result in bands in template-free lanes. In the absence of DNA and polymerase (Taq) the only helical structures are those of intermolecular primer binding (non-extension dimers) or intramolecular binding (hairpins). Ethidium bromide also weakly binds to single-stranded DNA and unbound primers can appear as small bands. Primer bands are particularly evident in gels stained with fluorescent dyes that bind more strongly to single-stranded DNA such as GelRed™.

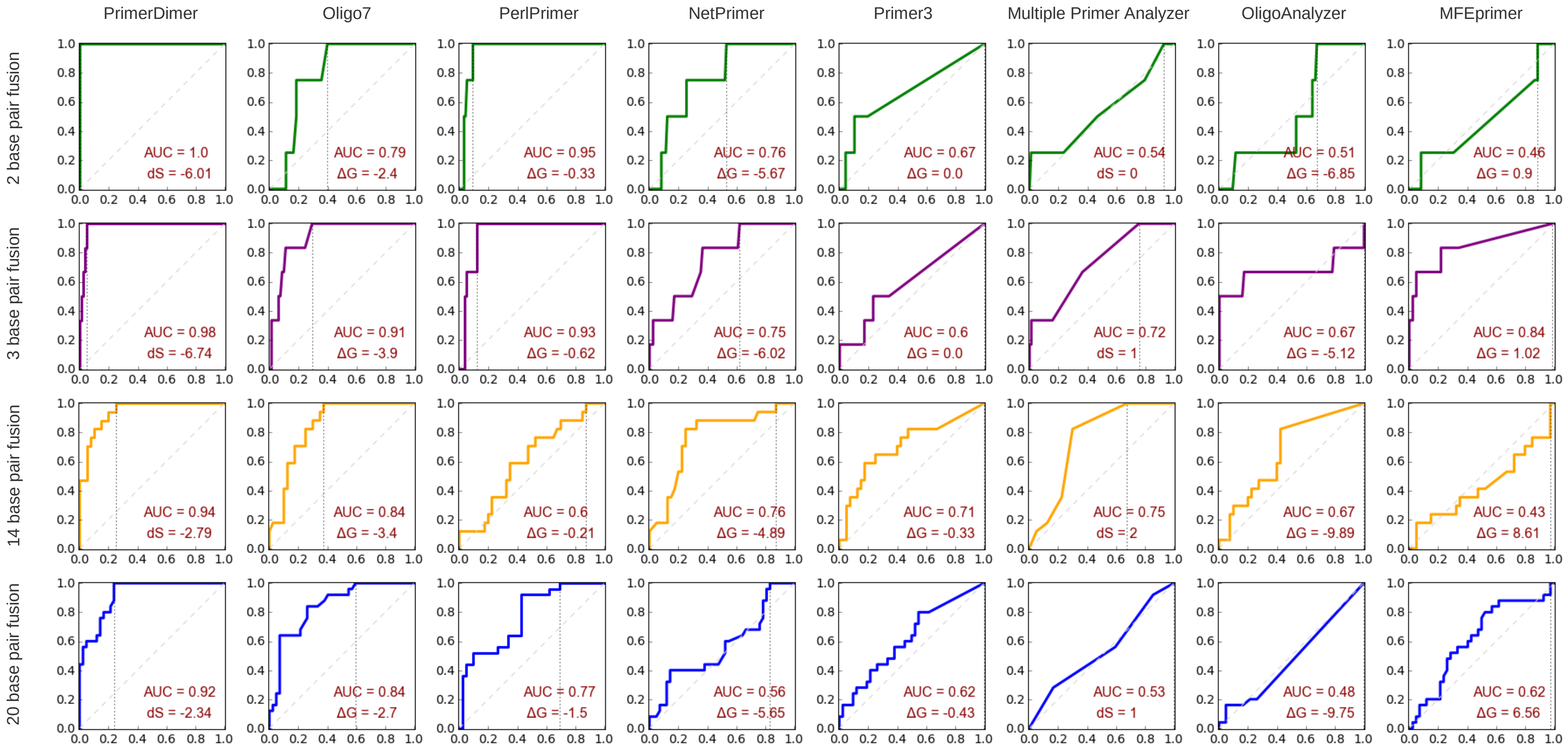

Supplementary Figure S7 | Receiver operating characteristic (ROC) curves depicting dimer prediction performance of PrimerROC and seven freely available primer design/analysis tools. Each curve includes accuracy of dimer algorithms measured by area under the curve (AUC), and dimer score (dS/ΔG) at the point where zero dimer-forming primer pairs are misclassified as dimer-free. PrimerROC out performs all other algorithms in every primer set—both in AUC and proportion of correctly classified primers at the dimer-free determination threshold. Y-axis = true-positive rate (sensitivity). X-axis = false-positive rate (1-specificity).

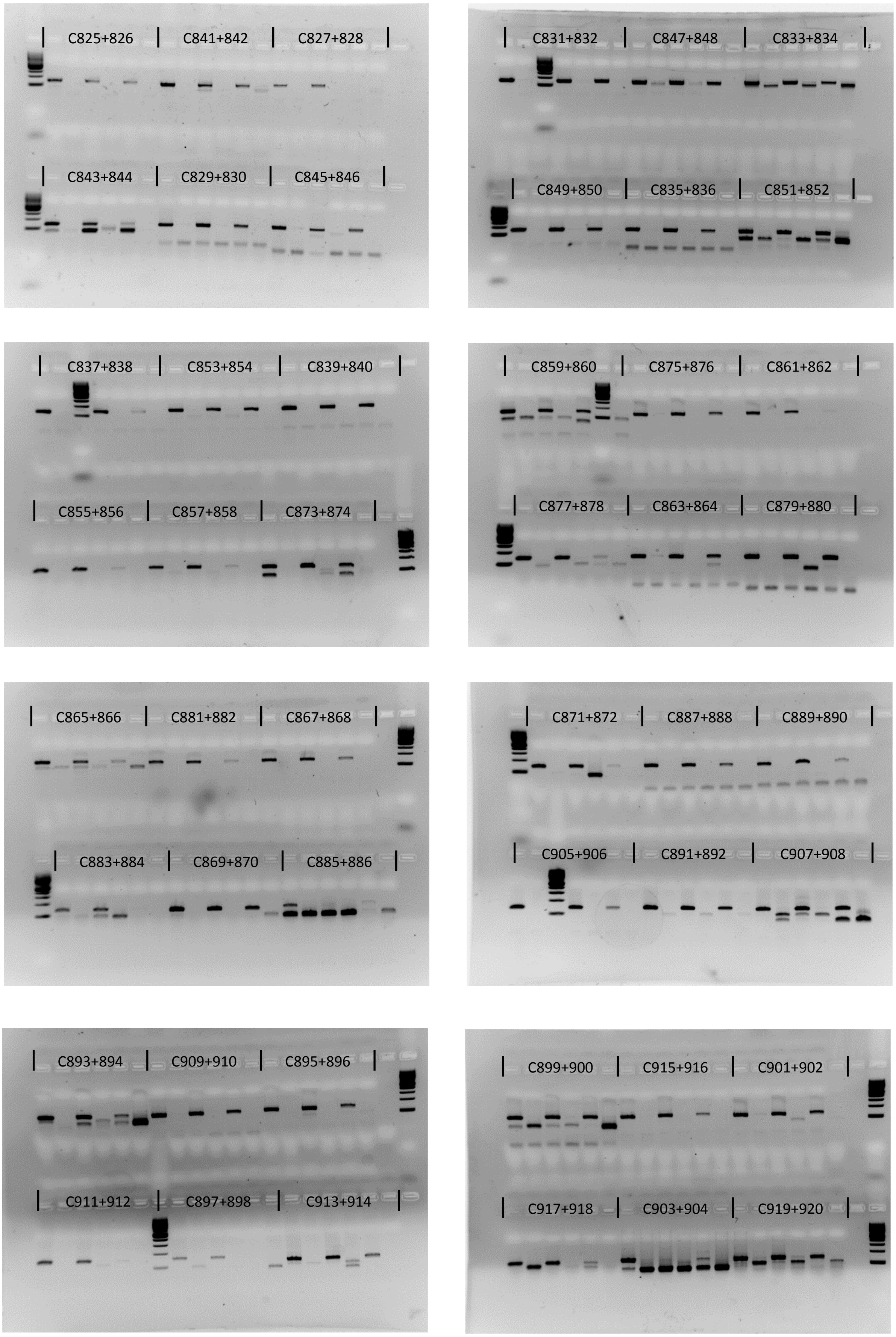

Supplementary Figure S8 | Gel electrophoresis images of 14 base pair fusion primer PCR products and dimer artefacts of altered conditions. Each primer pair includes the following three conditions from left to right: 1.5 mM TMAC; 1.5% formamide; and 4% formamide. First lane of each condition contains template and second lane is template free. Ladder is 100 bps. Stained with ethidium bromide.

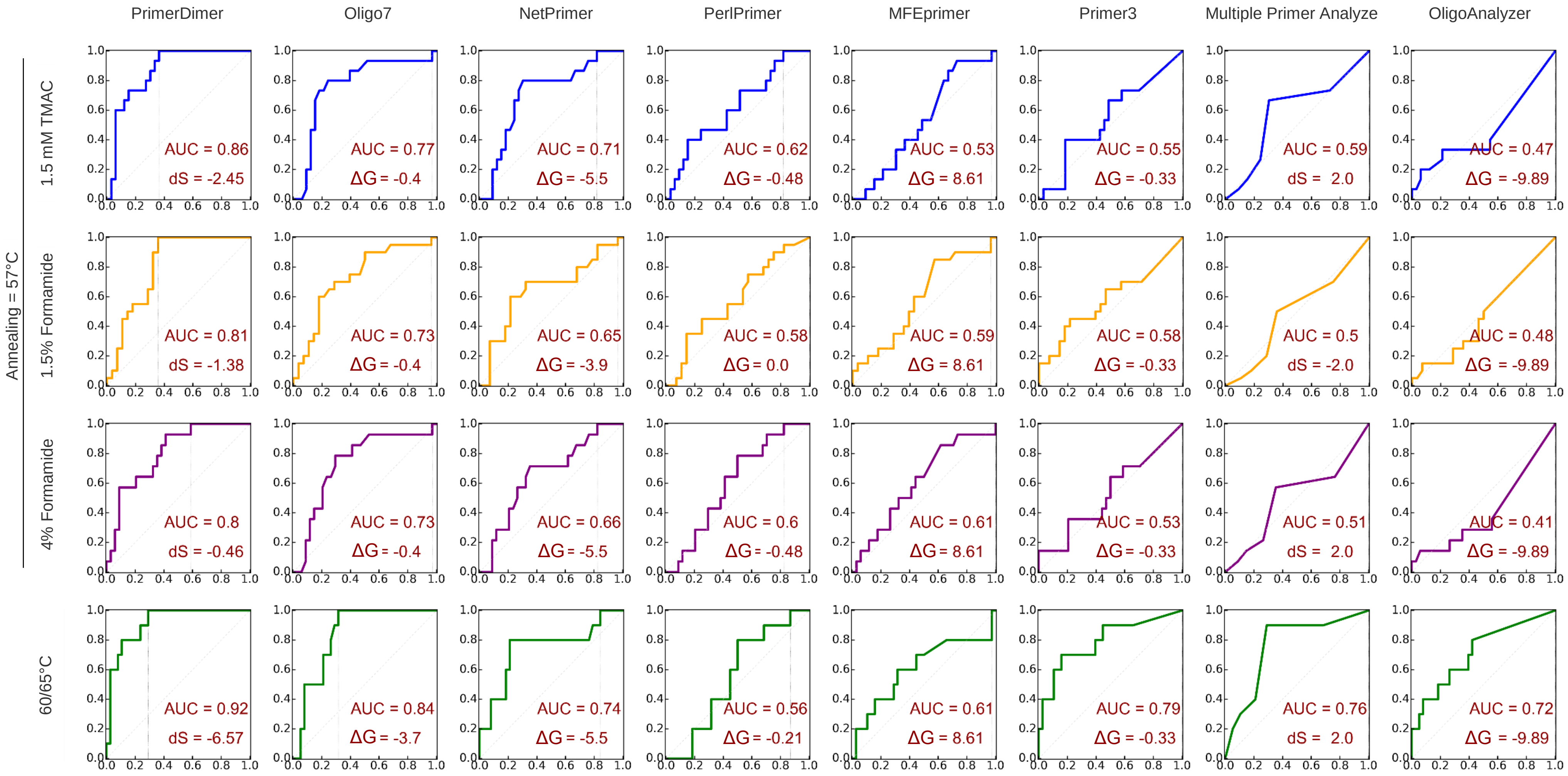

Supplementary Figure S9 | Receiver operating characteristic (ROC) curves depicting dimer prediction performance of PrimerROC and seven freely available dimer analysis tools under varying PCR conditions. Each curve includes accuracy of dimer algorithms measured by area under the curve (AUC), and dimer score (dS/ ΔG) at the point where zero dimer-forming primer pairs are misclassified as dimer-free. PrimerROC out-performs all other algorithms in every primer set—both in AUC and proportion of correctly classified primers at the dimer-free determination threshold. Y-axis = true-positive rate (sensitivity). X-axis = false-positive rate (1-specificity). These analyses were performed on 48 primers pairs of the 14 base pair fusion set.

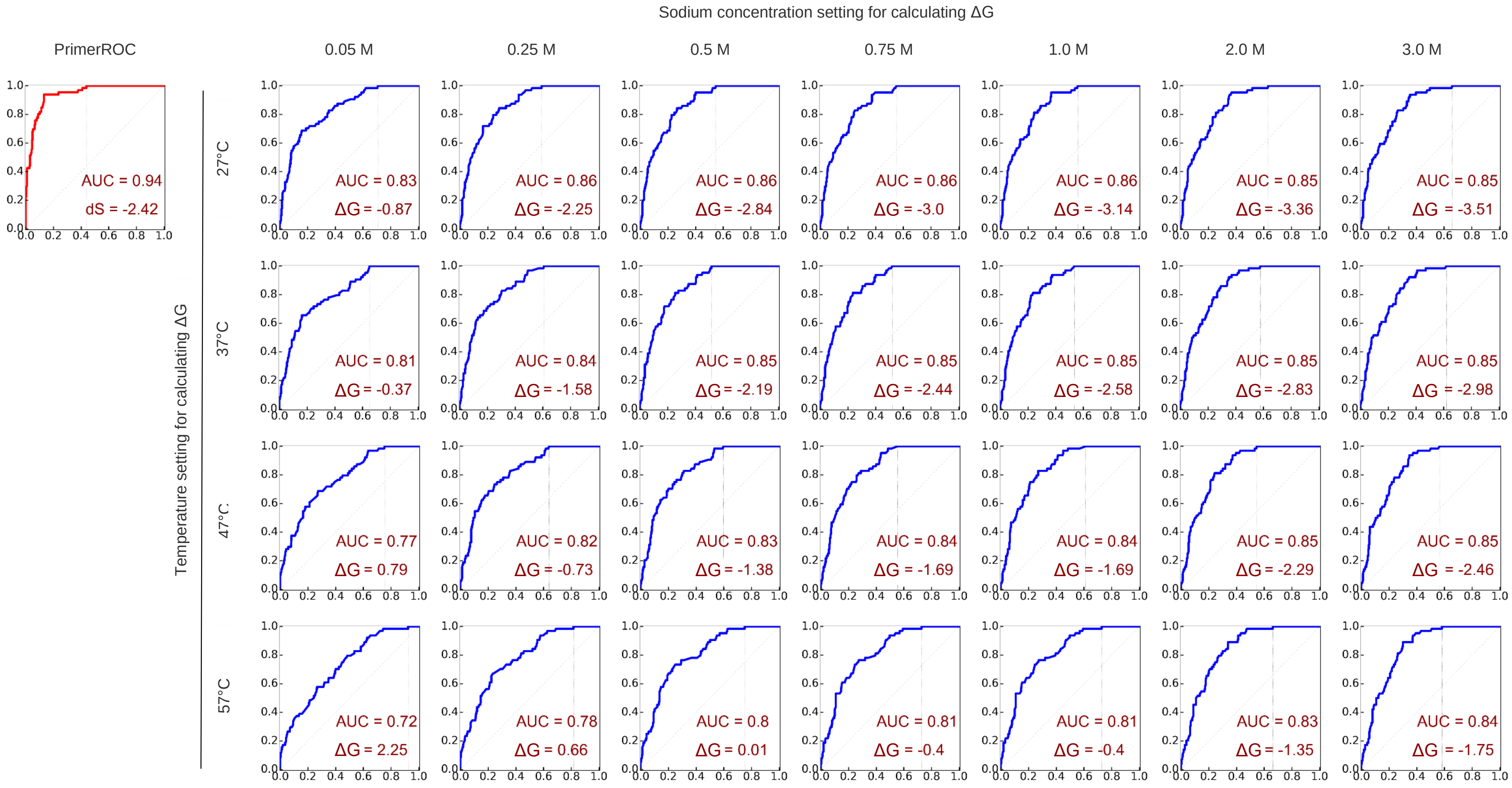

Supplementary Figure S10 | Receiver operating characteristic (ROC) curves depicting dimer prediction performance of ΔG using varying temperature and sodium concentration values. ROC curves were created using the combined set of all fusion primers with stringent dimer classification (all bands originally classified as ambiguous were reclassified as dimer-forming). Each curve includes dimer prediction accuracy measured by area under the curve (AUC), and dimer score (dS/ΔG) at the point where zero dimer-forming primer pairs are misclassified as dimer-free. Altering temperature and sodium concentration parameters when calculating ΔG can greatly impact its predictive accuracy. However, the standard 37°C with 1 M sodium approaches maximum accuracy, with a lower sodium concentration of 0.5 M giving slightly better performance. PrimerROC/PrimerDimer results in substantially greater prediction accuracy over ΔG alone. Y-axis = true-positive rate (sensitivity). X-axis = false-positive rate (1-specificity).

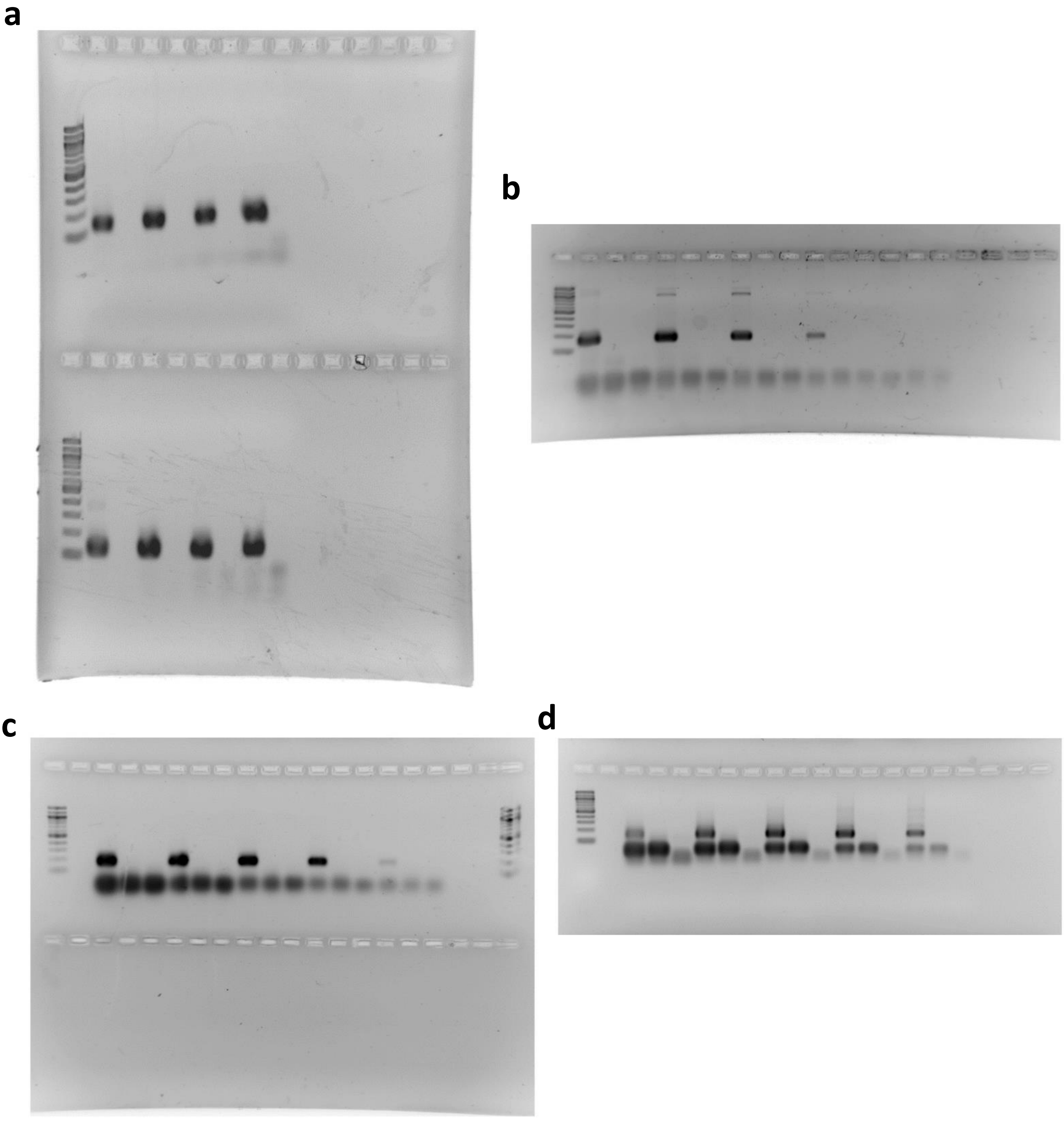

Supplementary Figure S11 | Full gel electrophoresis images of multiplex assays at various primer concentrations. The first lane for each concentration contains template, the second is template-free. The third lane for each concentration in **b-d** is template-free and polymerase-free. **(a)** Top half of gel is neurological bisulfite PCR pool 1 (53 primer pairs) with 0.25, 0.5, 1 and 2 µM total primer concentration from left to right. Bottom half is neurological bisulfite PCR pool 2 (45 primer pairs) with 0.25, 0.5, 1 and 2 µM total primer concentration from left to right. **(b)** Somatic mutations in cancer panel genomic PCR (44 primer pairs) with 10, 5, 2.5 and 1.25 µM total primer concentration from left to right. **(c)** SNPs in sugarcane panel genomic PCR (63 primer pairs) with 8, 4, 2, 1 and 0.5 µM total primer concentration from left to right. **(d)** Exemplar multiplex bisulfite assay where PrimerROC was not used and visible dimers formed under the same PCR conditions as the dimer-free assays in **a-c** (123 primer pairs) with 10, 5, 2.5, 1.25 and 0.625 µM total primer concentration from left to right. Ladder is 100 bps. All gels stained with ethidium bromide.

a

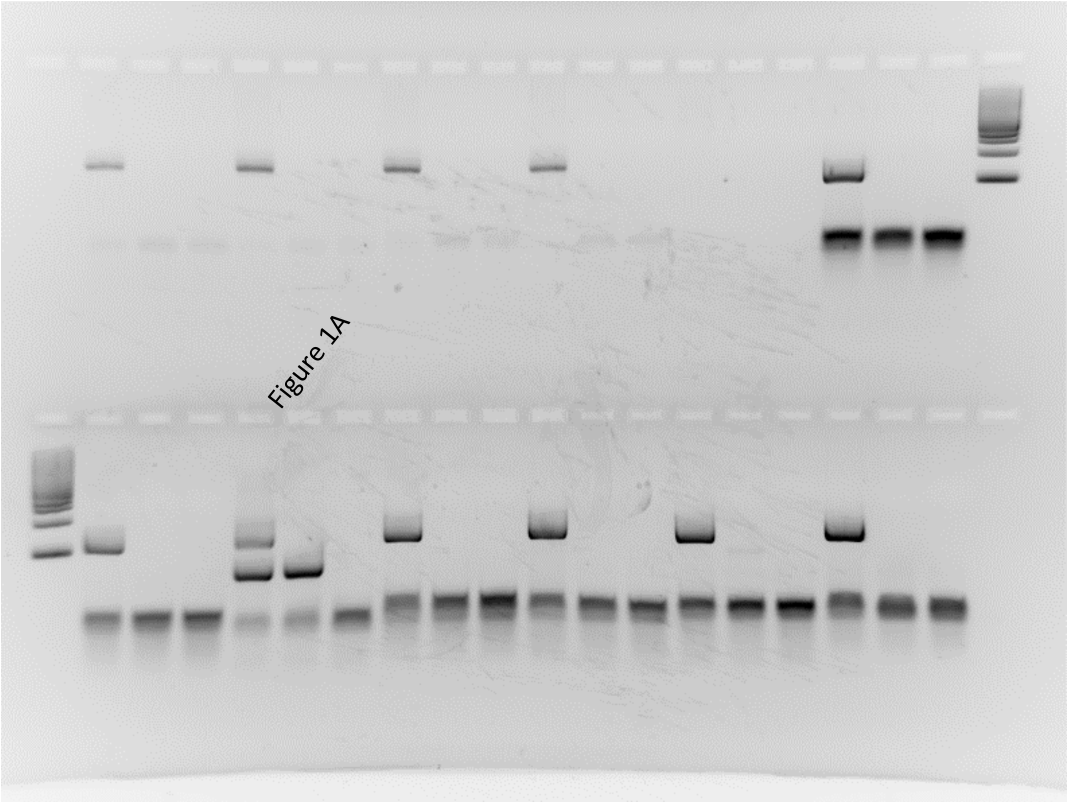

b

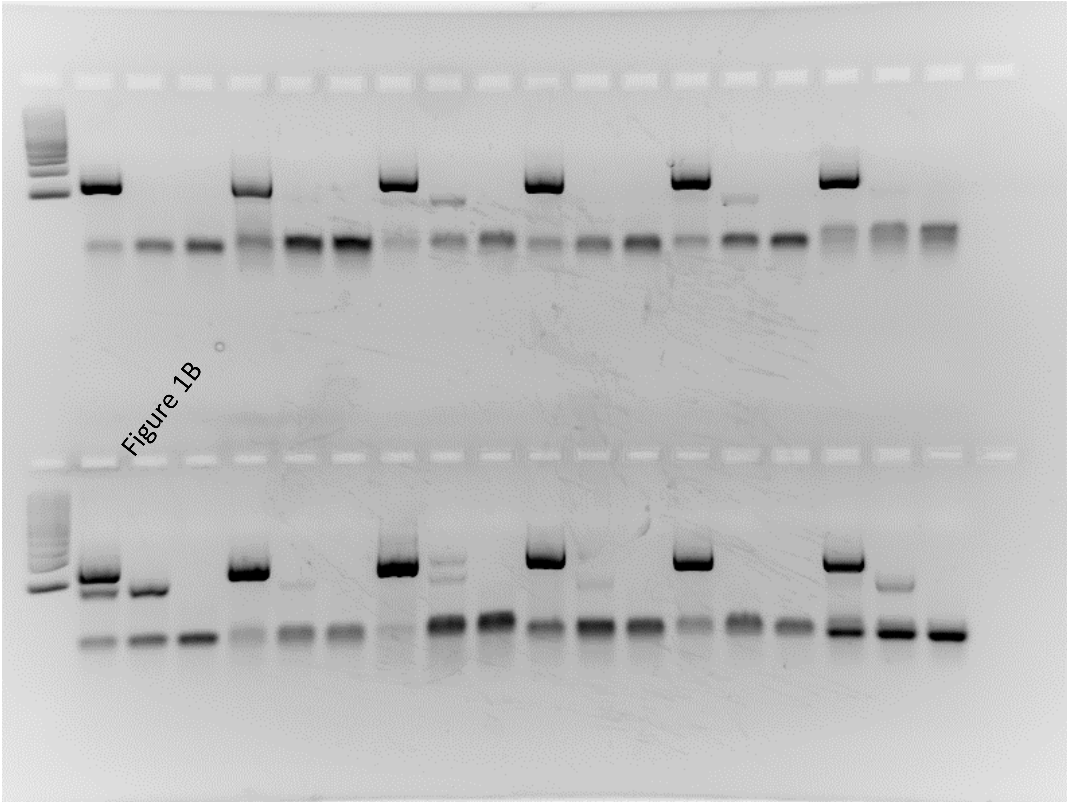

Supplementary Figure S12 | Full gel electrophoresis images of sequenced dimer products. The first lane for each primer pair contains template, the second is template-free and the third is template-free and polymerase-free. **(a)** Gel of primer pair set including dimer product displayed in Figure 1A. **(b)** Gel of primer pair set including dimer product displayed in Figure 1B. Ladder is 100 bps. Gels stained with GelRed™.
